# Search for invisible decays of the Higgs boson produced in association with a hadronically decaying vector boson in *pp* collisions at $\sqrt{s} = 8$ TeV with the ATLAS detector

**DOI:** 10.1140/epjc/s10052-015-3551-1

**Published:** 2015-07-18

**Authors:** G. Aad, B. Abbott, J. Abdallah, O. Abdinov, R. Aben, M. Abolins, O. S. AbouZeid, H. Abramowicz, H. Abreu, R. Abreu, Y. Abulaiti, B. S. Acharya, L. Adamczyk, D. L. Adams, J. Adelman, S. Adomeit, T. Adye, A. A. Affolder, T. Agatonovic-Jovin, J. A. Aguilar-Saavedra, S. P. Ahlen, F. Ahmadov, G. Aielli, H. Akerstedt, T. P. A. Åkesson, G. Akimoto, A. V. Akimov, G. L. Alberghi, J. Albert, S. Albrand, M. J. Alconada Verzini, M. Aleksa, I. N. Aleksandrov, C. Alexa, G. Alexander, T. Alexopoulos, M. Alhroob, G. Alimonti, L. Alio, J. Alison, S. P. Alkire, B. M. M. Allbrooke, P. P. Allport, A. Aloisio, A. Alonso, F. Alonso, C. Alpigiani, A. Altheimer, B. Alvarez Gonzalez, D. Álvarez Piqueras, M. G. Alviggi, B. T. Amadio, K. Amako, Y. Amaral Coutinho, C. Amelung, D. Amidei, S. P. Amor Dos Santos, A. Amorim, S. Amoroso, N. Amram, G. Amundsen, C. Anastopoulos, L. S. Ancu, N. Andari, T. Andeen, C. F. Anders, G. Anders, J. K. Anders, K. J. Anderson, A. Andreazza, V. Andrei, S. Angelidakis, I. Angelozzi, P. Anger, A. Angerami, F. Anghinolfi, A. V. Anisenkov, N. Anjos, A. Annovi, M. Antonelli, A. Antonov, J. Antos, F. Anulli, M. Aoki, L. Aperio Bella, G. Arabidze, Y. Arai, J. P. Araque, A. T. H. Arce, F. A Arduh, J-F. Arguin, S. Argyropoulos, M. Arik, A. J. Armbruster, O. Arnaez, V. Arnal, H. Arnold, M. Arratia, O. Arslan, A. Artamonov, G. Artoni, S. Asai, N. Asbah, A. Ashkenazi, B. Åsman, L. Asquith, K. Assamagan, R. Astalos, M. Atkinson, N. B. Atlay, B. Auerbach, K. Augsten, M. Aurousseau, G. Avolio, B. Axen, M. K. Ayoub, G. Azuelos, M. A. Baak, A. E. Baas, C. Bacci, H. Bachacou, K. Bachas, M. Backes, M. Backhaus, E. Badescu, P. Bagiacchi, P. Bagnaia, Y. Bai, T. Bain, J. T. Baines, O. K. Baker, P. Balek, T. Balestri, F. Balli, E. Banas, Sw. Banerjee, A. A. E. Bannoura, H. S. Bansil, L. Barak, S. P. Baranov, E. L. Barberio, D. Barberis, M. Barbero, T. Barillari, M. Barisonzi, T. Barklow, N. Barlow, S. L. Barnes, B. M. Barnett, R. M. Barnett, Z. Barnovska, A. Baroncelli, G. Barone, A. J. Barr, F. Barreiro, J. Barreiro Guimarães da Costa, R. Bartoldus, A. E. Barton, P. Bartos, A. Bassalat, A. Basye, R. L. Bates, S. J. Batista, J. R. Batley, M. Battaglia, M. Bauce, F. Bauer, H. S. Bawa, J. B. Beacham, M. D. Beattie, T. Beau, P. H. Beauchemin, R. Beccherle, P. Bechtle, H. P. Beck, K. Becker, M. Becker, S. Becker, M. Beckingham, C. Becot, A. J. Beddall, A. Beddall, V. A. Bednyakov, C. P. Bee, L. J. Beemster, T. A. Beermann, M. Begel, J. K. Behr, C. Belanger-Champagne, P. J. Bell, W. H. Bell, G. Bella, L. Bellagamba, A. Bellerive, M. Bellomo, K. Belotskiy, O. Beltramello, O. Benary, D. Benchekroun, M. Bender, K. Bendtz, N. Benekos, Y. Benhammou, E. Benhar Noccioli, J. A. Benitez Garcia, D. P. Benjamin, J. R. Bensinger, S. Bentvelsen, L. Beresford, M. Beretta, D. Berge, E. Bergeaas Kuutmann, N. Berger, F. Berghaus, J. Beringer, C. Bernard, N. R. Bernard, C. Bernius, F. U. Bernlochner, T. Berry, P. Berta, C. Bertella, G. Bertoli, F. Bertolucci, C. Bertsche, D. Bertsche, M. I. Besana, G. J. Besjes, O. Bessidskaia Bylund, M. Bessner, N. Besson, C. Betancourt, S. Bethke, A. J. Bevan, W. Bhimji, R. M. Bianchi, L. Bianchini, M. Bianco, O. Biebel, S. P. Bieniek, M. Biglietti, J. Bilbao De Mendizabal, H. Bilokon, M. Bindi, S. Binet, A. Bingul, C. Bini, C. W. Black, J. E. Black, K. M. Black, D. Blackburn, R. E. Blair, J.-B. Blanchard, J. E. Blanco, T. Blazek, I. Bloch, C. Blocker, W. Blum, U. Blumenschein, G. J. Bobbink, V. S. Bobrovnikov, S. S. Bocchetta, A. Bocci, C. Bock, M. Boehler, J. A. Bogaerts, A. G. Bogdanchikov, C. Bohm, V. Boisvert, T. Bold, V. Boldea, A. S. Boldyrev, M. Bomben, M. Bona, M. Boonekamp, A. Borisov, G. Borissov, S. Borroni, J. Bortfeldt, V. Bortolotto, K. Bos, D. Boscherini, M. Bosman, J. Boudreau, J. Bouffard, E. V. Bouhova-Thacker, D. Boumediene, C. Bourdarios, N. Bousson, A. Boveia, J. Boyd, I. R. Boyko, I. Bozic, J. Bracinik, A. Brandt, G. Brandt, O. Brandt, U. Bratzler, B. Brau, J. E. Brau, H. M. Braun, S. F. Brazzale, K. Brendlinger, A. J. Brennan, L. Brenner, R. Brenner, S. Bressler, K. Bristow, T. M. Bristow, D. Britton, D. Britzger, F. M. Brochu, I. Brock, R. Brock, J. Bronner, G. Brooijmans, T. Brooks, W. K. Brooks, J. Brosamer, E. Brost, J. Brown, P. A. Bruckman de Renstrom, D. Bruncko, R. Bruneliere, A. Bruni, G. Bruni, M. Bruschi, L. Bryngemark, T. Buanes, Q. Buat, P. Buchholz, A. G. Buckley, S. I. Buda, I. A. Budagov, F. Buehrer, L. Bugge, M. K. Bugge, O. Bulekov, D. Bullock, H. Burckhart, S. Burdin, B. Burghgrave, S. Burke, I. Burmeister, E. Busato, D. Büscher, V. Büscher, P. Bussey, C. P. Buszello, J. M. Butler, A. I. Butt, C. M. Buttar, J. M. Butterworth, P. Butti, W. Buttinger, A. Buzatu, R. Buzykaev, S. Cabrera Urbán, D. Caforio, V. M. Cairo, O. Cakir, P. Calafiura, A. Calandri, G. Calderini, P. Calfayan, L. P. Caloba, D. Calvet, S. Calvet, R. Camacho Toro, S. Camarda, P. Camarri, D. Cameron, L. M. Caminada, R. Caminal Armadans, S. Campana, M. Campanelli, A. Campoverde, V. Canale, A. Canepa, M. Cano Bret, J. Cantero, R. Cantrill, T. Cao, M. D. M. Capeans Garrido, I. Caprini, M. Caprini, M. Capua, R. Caputo, R. Cardarelli, T. Carli, G. Carlino, L. Carminati, S. Caron, E. Carquin, G. D. Carrillo-Montoya, J. R. Carter, J. Carvalho, D. Casadei, M. P. Casado, M. Casolino, E. Castaneda-Miranda, A. Castelli, V. Castillo Gimenez, N. F. Castro, P. Catastini, A. Catinaccio, J. R. Catmore, A. Cattai, J. Caudron, V. Cavaliere, D. Cavalli, M. Cavalli-Sforza, V. Cavasinni, F. Ceradini, B. C. Cerio, K. Cerny, A. S. Cerqueira, A. Cerri, L. Cerrito, F. Cerutti, M. Cerv, A. Cervelli, S. A. Cetin, A. Chafaq, D. Chakraborty, I. Chalupkova, P. Chang, B. Chapleau, J. D. Chapman, D. G. Charlton, C. C. Chau, C. A. Chavez Barajas, S. Cheatham, A. Chegwidden, S. Chekanov, S. V. Chekulaev, G. A. Chelkov, M. A. Chelstowska, C. Chen, H. Chen, K. Chen, L. Chen, S. Chen, X. Chen, Y. Chen, H. C. Cheng, Y. Cheng, A. Cheplakov, E. Cheremushkina, R. Cherkaoui El Moursli, V. Chernyatin, E. Cheu, L. Chevalier, V. Chiarella, J. T. Childers, G. Chiodini, A. S. Chisholm, R. T. Chislett, A. Chitan, M. V. Chizhov, K. Choi, S. Chouridou, B. K. B. Chow, V. Christodoulou, D. Chromek-Burckhart, M. L. Chu, J. Chudoba, A. J. Chuinard, J. J. Chwastowski, L. Chytka, G. Ciapetti, A. K. Ciftci, D. Cinca, V. Cindro, I. A. Cioara, A. Ciocio, Z. H. Citron, M. Ciubancan, A. Clark, B. L. Clark, B. L. Clark, P. J. Clark, R. N. Clarke, W. Cleland, C. Clement, Y. Coadou, M. Cobal, A. Coccaro, J. Cochran, L. Coffey, J. G. Cogan, B. Cole, S. Cole, A. P. Colijn, J. Collot, T. Colombo, G. Compostella, P. Conde Muiño, E. Coniavitis, S. H. Connell, I. A. Connelly, S. M. Consonni, V. Consorti, S. Constantinescu, C. Conta, G. Conti, F. Conventi, M. Cooke, B. D. Cooper, A. M. Cooper-Sarkar, T. Cornelissen, M. Corradi, F. Corriveau, A. Corso-Radu, A. Cortes-Gonzalez, G. Cortiana, G. Costa, M. J. Costa, D. Costanzo, D. Côté, G. Cottin, G. Cowan, B. E. Cox, K. Cranmer, G. Cree, S. Crépé-Renaudin, F. Crescioli, W. A. Cribbs, M. Crispin Ortuzar, M. Cristinziani, V. Croft, G. Crosetti, T. Cuhadar Donszelmann, J. Cummings, M. Curatolo, C. Cuthbert, H. Czirr, P. Czodrowski, S. D’Auria, M. D’Onofrio, M. J. Da Cunha Sargedas De Sousa, C. Da Via, W. Dabrowski, A. Dafinca, T. Dai, O. Dale, F. Dallaire, C. Dallapiccola, M. Dam, J. R. Dandoy, N. P. Dang, A. C. Daniells, M. Danninger, M. Dano Hoffmann, V. Dao, G. Darbo, S. Darmora, J. Dassoulas, A. Dattagupta, W. Davey, C. David, T. Davidek, E. Davies, M. Davies, P. Davison, Y. Davygora, E. Dawe, I. Dawson, R. K. Daya-Ishmukhametova, K. De, R. de Asmundis, S. De Castro, S. De Cecco, N. De Groot, P. de Jong, H. De la Torre, F. De Lorenzi, L. De Nooij, D. De Pedis, A. De Salvo, U. De Sanctis, A. De Santo, J. B. De Vivie De Regie, W. J. Dearnaley, R. Debbe, C. Debenedetti, D. V. Dedovich, I. Deigaard, J. Del Peso, T. Del Prete, D. Delgove, F. Deliot, C. M. Delitzsch, M. Deliyergiyev, A. Dell’Acqua, L. Dell’Asta, M. Dell’Orso, M. Della Pietra, D. della Volpe, M. Delmastro, P. A. Delsart, C. Deluca, D. A. DeMarco, S. Demers, M. Demichev, A. Demilly, S. P. Denisov, D. Derendarz, J. E. Derkaoui, F. Derue, P. Dervan, K. Desch, C. Deterre, P. O. Deviveiros, A. Dewhurst, S. Dhaliwal, A. Di Ciaccio, L. Di Ciaccio, A. Di Domenico, C. Di Donato, A. Di Girolamo, B. Di Girolamo, A. Di Mattia, B. Di Micco, R. Di Nardo, A. Di Simone, R. Di Sipio, D. Di Valentino, C. Diaconu, M. Diamond, F. A. Dias, M. A. Diaz, E. B. Diehl, J. Dietrich, S. Diglio, A. Dimitrievska, J. Dingfelder, F. Dittus, F. Djama, T. Djobava, J. I. Djuvsland, M. A. B. do Vale, D. Dobos, M. Dobre, C. Doglioni, T. Dohmae, J. Dolejsi, Z. Dolezal, B. A. Dolgoshein, M. Donadelli, S. Donati, P. Dondero, J. Donini, J. Dopke, A. Doria, M. T. Dova, A. T. Doyle, E. Drechsler, M. Dris, E. Dubreuil, E. Duchovni, G. Duckeck, O. A. Ducu, D. Duda, A. Dudarev, L. Duflot, L. Duguid, M. Dührssen, M. Dunford, H. Duran Yildiz, M. Düren, A. Durglishvili, D. Duschinger, M. Dyndal, C. Eckardt, K. M. Ecker, R. C. Edgar, W. Edson, N. C. Edwards, W. Ehrenfeld, T. Eifert, G. Eigen, K. Einsweiler, T. Ekelof, M. El Kacimi, M. Ellert, S. Elles, F. Ellinghaus, A. A. Elliot, N. Ellis, J. Elmsheuser, M. Elsing, D. Emeliyanov, Y. Enari, O. C. Endner, M. Endo, R. Engelmann, J. Erdmann, A. Ereditato, G. Ernis, J. Ernst, M. Ernst, S. Errede, E. Ertel, M. Escalier, H. Esch, C. Escobar, B. Esposito, A. I. Etienvre, E. Etzion, H. Evans, A. Ezhilov, L. Fabbri, G. Facini, R. M. Fakhrutdinov, S. Falciano, R. J. Falla, J. Faltova, Y. Fang, M. Fanti, A. Farbin, A. Farilla, T. Farooque, S. Farrell, S. M. Farrington, P. Farthouat, F. Fassi, P. Fassnacht, D. Fassouliotis, M. Faucci Giannelli, A. Favareto, L. Fayard, P. Federic, O. L. Fedin, W. Fedorko, S. Feigl, L. Feligioni, C. Feng, E. J. Feng, H. Feng, A. B. Fenyuk, P. Fernandez Martinez, S. Fernandez Perez, S. Ferrag, J. Ferrando, A. Ferrari, P. Ferrari, R. Ferrari, D. E. Ferreira de Lima, A. Ferrer, D. Ferrere, C. Ferretti, A. Ferretto Parodi, M. Fiascaris, F. Fiedler, A. Filipčič, M. Filipuzzi, F. Filthaut, M. Fincke-Keeler, K. D. Finelli, M. C. N. Fiolhais, L. Fiorini, A. Firan, A. Fischer, C. Fischer, J. Fischer, W. C. Fisher, E. A. Fitzgerald, M. Flechl, I. Fleck, P. Fleischmann, S. Fleischmann, G. T. Fletcher, G. Fletcher, T. Flick, A. Floderus, L. R. Flores Castillo, M. J. Flowerdew, A. Formica, A. Forti, D. Fournier, H. Fox, S. Fracchia, P. Francavilla, M. Franchini, D. Francis, L. Franconi, M. Franklin, M. Fraternali, D. Freeborn, S. T. French, F. Friedrich, D. Froidevaux, J. A. Frost, C. Fukunaga, E. Fullana Torregrosa, B. G. Fulsom, J. Fuster, C. Gabaldon, O. Gabizon, A. Gabrielli, A. Gabrielli, S. Gadatsch, S. Gadomski, G. Gagliardi, P. Gagnon, C. Galea, B. Galhardo, E. J. Gallas, B. J. Gallop, P. Gallus, G. Galster, K. K. Gan, J. Gao, Y. Gao, Y. S. Gao, F. M. Garay Walls, F. Garberson, C. García, J. E. García Navarro, M. Garcia-Sciveres, R. W. Gardner, N. Garelli, V. Garonne, C. Gatti, A. Gaudiello, G. Gaudio, B. Gaur, L. Gauthier, P. Gauzzi, I. L. Gavrilenko, C. Gay, G. Gaycken, E. N. Gazis, P. Ge, Z. Gecse, C. N. P. Gee, D. A. A. Geerts, Ch. Geich-Gimbel, M. P. Geisler, C. Gemme, M. H. Genest, S. Gentile, M. George, S. George, D. Gerbaudo, A. Gershon, H. Ghazlane, B. Giacobbe, S. Giagu, V. Giangiobbe, P. Giannetti, B. Gibbard, S. M. Gibson, M. Gilchriese, T. P. S. Gillam, D. Gillberg, G. Gilles, D. M. Gingrich, N. Giokaris, M. P. Giordani, F. M. Giorgi, F. M. Giorgi, P. F. Giraud, P. Giromini, D. Giugni, C. Giuliani, M. Giulini, B. K. Gjelsten, S. Gkaitatzis, I. Gkialas, E. L. Gkougkousis, L. K. Gladilin, C. Glasman, J. Glatzer, P. C. F. Glaysher, A. Glazov, G. L. Glonti, M. Goblirsch-Kolb, J. R. Goddard, J. Godlewski, S. Goldfarb, T. Golling, D. Golubkov, A. Gomes, R. Gonçalo, J. Goncalves Pinto Firmino Da Costa, L. Gonella, S. González de la Hoz, G. Gonzalez Parra, S. Gonzalez-Sevilla, L. Goossens, P. A. Gorbounov, H. A. Gordon, I. Gorelov, B. Gorini, E. Gorini, A. Gorišek, E. Gornicki, A. T. Goshaw, C. Gössling, M. I. Gostkin, D. Goujdami, A. G. Goussiou, N. Govender, H. M. X. Grabas, L. Graber, I. Grabowska-Bold, P. Grafström, K-J. Grahn, J. Gramling, E. Gramstad, S. Grancagnolo, V. Grassi, V. Gratchev, H. M. Gray, E. Graziani, Z. D. Greenwood, K. Gregersen, I. M. Gregor, P. Grenier, J. Griffiths, A. A. Grillo, K. Grimm, S. Grinstein, Ph. Gris, J.-F. Grivaz, J. P. Grohs, A. Grohsjean, E. Gross, J. Grosse-Knetter, G. C. Grossi, Z. J. Grout, L. Guan, J. Guenther, F. Guescini, D. Guest, O. Gueta, E. Guido, T. Guillemin, S. Guindon, U. Gul, C. Gumpert, J. Guo, S. Gupta, P. Gutierrez, N. G. Gutierrez Ortiz, C. Gutschow, C. Guyot, C. Gwenlan, C. B. Gwilliam, A. Haas, C. Haber, H. K. Hadavand, N. Haddad, P. Haefner, S. Hageböck, Z. Hajduk, H. Hakobyan, M. Haleem, J. Haley, D. Hall, G. Halladjian, G. D. Hallewell, K. Hamacher, P. Hamal, K. Hamano, M. Hamer, A. Hamilton, S. Hamilton, G. N. Hamity, P. G. Hamnett, L. Han, K. Hanagaki, K. Hanawa, M. Hance, P. Hanke, R. Hanna, J. B. Hansen, J. D. Hansen, M. C. Hansen, P. H. Hansen, K. Hara, A. S. Hard, T. Harenberg, F. Hariri, S. Harkusha, R. D. Harrington, P. F. Harrison, F. Hartjes, M. Hasegawa, S. Hasegawa, Y. Hasegawa, A. Hasib, S. Hassani, S. Haug, R. Hauser, L. Hauswald, M. Havranek, C. M. Hawkes, R. J. Hawkings, A. D. Hawkins, T. Hayashi, D. Hayden, C. P. Hays, J. M. Hays, H. S. Hayward, S. J. Haywood, S. J. Head, T. Heck, V. Hedberg, L. Heelan, S. Heim, T. Heim, B. Heinemann, L. Heinrich, J. Hejbal, L. Helary, S. Hellman, D. Hellmich, C. Helsens, J. Henderson, R. C. W. Henderson, Y. Heng, C. Hengler, A. Henrichs, A. M. Henriques Correia, S. Henrot-Versille, G. H. Herbert, Y. Hernández Jiménez, R. Herrberg-Schubert, G. Herten, R. Hertenberger, L. Hervas, G. G. Hesketh, N. P. Hessey, J. W. Hetherly, R. Hickling, E. Higón-Rodriguez, E. Hill, J. C. Hill, K. H. Hiller, S. J. Hillier, I. Hinchliffe, E. Hines, R. R. Hinman, M. Hirose, D. Hirschbuehl, J. Hobbs, N. Hod, M. C. Hodgkinson, P. Hodgson, A. Hoecker, M. R. Hoeferkamp, F. Hoenig, M. Hohlfeld, D. Hohn, T. R. Holmes, T. M. Hong, L. Hooft van Huysduynen, W. H. Hopkins, Y. Horii, A. J. Horton, J-Y. Hostachy, S. Hou, A. Hoummada, J. Howard, J. Howarth, M. Hrabovsky, I. Hristova, J. Hrivnac, T. Hryn’ova, A. Hrynevich, C. Hsu, P. J. Hsu, S.-C. Hsu, D. Hu, Q. Hu, X. Hu, Y. Huang, Z. Hubacek, F. Hubaut, F. Huegging, T. B. Huffman, E. W. Hughes, G. Hughes, M. Huhtinen, T. A. Hülsing, N. Huseynov, J. Huston, J. Huth, G. Iacobucci, G. Iakovidis, I. Ibragimov, L. Iconomidou-Fayard, E. Ideal, Z. Idrissi, P. Iengo, O. Igonkina, T. Iizawa, Y. Ikegami, K. Ikematsu, M. Ikeno, Y. Ilchenko, D. Iliadis, N. Ilic, Y. Inamaru, T. Ince, P. Ioannou, M. Iodice, K. Iordanidou, V. Ippolito, A. Irles Quiles, C. Isaksson, M. Ishino, M. Ishitsuka, R. Ishmukhametov, C. Issever, S. Istin, J. M. Iturbe Ponce, R. Iuppa, J. Ivarsson, W. Iwanski, H. Iwasaki, J. M. Izen, V. Izzo, S. Jabbar, B. Jackson, M. Jackson, P. Jackson, M. R. Jaekel, V. Jain, K. Jakobs, S. Jakobsen, T. Jakoubek, J. Jakubek, D. O. Jamin, D. K. Jana, E. Jansen, R. W. Jansky, J. Janssen, M. Janus, G. Jarlskog, N. Javadov, T. Javůrek, L. Jeanty, J. Jejelava, G.-Y. Jeng, D. Jennens, P. Jenni, J. Jentzsch, C. Jeske, S. Jézéquel, H. Ji, J. Jia, Y. Jiang, S. Jiggins, J. Jimenez Pena, S. Jin, A. Jinaru, O. Jinnouchi, M. D. Joergensen, P. Johansson, K. A. Johns, K. Jon-And, G. Jones, R. W. L. Jones, T. J. Jones, J. Jongmanns, P. M. Jorge, K. D. Joshi, J. Jovicevic, X. Ju, C. A. Jung, P. Jussel, A. Juste Rozas, M. Kaci, A. Kaczmarska, M. Kado, H. Kagan, M. Kagan, S. J. Kahn, E. Kajomovitz, C. W. Kalderon, S. Kama, A. Kamenshchikov, N. Kanaya, M. Kaneda, S. Kaneti, V. A. Kantserov, J. Kanzaki, B. Kaplan, A. Kapliy, D. Kar, K. Karakostas, A. Karamaoun, N. Karastathis, M. J. Kareem, M. Karnevskiy, S. N. Karpov, Z. M. Karpova, K. Karthik, V. Kartvelishvili, A. N. Karyukhin, L. Kashif, R. D. Kass, A. Kastanas, Y. Kataoka, A. Katre, J. Katzy, K. Kawagoe, T. Kawamoto, G. Kawamura, S. Kazama, V. F. Kazanin, M. Y. Kazarinov, R. Keeler, R. Kehoe, J. S. Keller, J. J. Kempster, H. Keoshkerian, O. Kepka, B. P. Kerševan, S. Kersten, R. A. Keyes, F. Khalil-zada, H. Khandanyan, A. Khanov, A. G. Kharlamov, T. J. Khoo, V. Khovanskiy, E. Khramov, J. Khubua, H. Y. Kim, H. Kim, S. H. Kim, Y. Kim, N. Kimura, O. M. Kind, B. T. King, M. King, R. S. B. King, S. B. King, J. Kirk, A. E. Kiryunin, T. Kishimoto, D. Kisielewska, F. Kiss, K. Kiuchi, O. Kivernyk, E. Kladiva, M. H. Klein, M. Klein, U. Klein, K. Kleinknecht, P. Klimek, A. Klimentov, R. Klingenberg, J. A. Klinger, T. Klioutchnikova, E.-E. Kluge, P. Kluit, S. Kluth, E. Kneringer, E. B. F. G. Knoops, A. Knue, A. Kobayashi, D. Kobayashi, T. Kobayashi, M. Kobel, M. Kocian, P. Kodys, T. Koffas, E. Koffeman, L. A. Kogan, S. Kohlmann, Z. Kohout, T. Kohriki, T. Koi, H. Kolanoski, I. Koletsou, A. A. Komar, Y. Komori, T. Kondo, N. Kondrashova, K. Köneke, A. C. König, S. König, T. Kono, R. Konoplich, N. Konstantinidis, R. Kopeliansky, S. Koperny, L. Köpke, A. K. Kopp, K. Korcyl, K. Kordas, A. Korn, A. A. Korol, I. Korolkov, E. V. Korolkova, O. Kortner, S. Kortner, T. Kosek, V. V. Kostyukhin, V. M. Kotov, A. Kotwal, A. Kourkoumeli-Charalampidi, C. Kourkoumelis, V. Kouskoura, A. Koutsman, R. Kowalewski, T. Z. Kowalski, W. Kozanecki, A. S. Kozhin, V. A. Kramarenko, G. Kramberger, D. Krasnopevtsev, M. W. Krasny, A. Krasznahorkay, J. K. Kraus, A. Kravchenko, S. Kreiss, M. Kretz, J. Kretzschmar, K. Kreutzfeldt, P. Krieger, K. Krizka, K. Kroeninger, H. Kroha, J. Kroll, J. Kroseberg, J. Krstic, U. Kruchonak, H. Krüger, N. Krumnack, Z. V. Krumshteyn, A. Kruse, M. C. Kruse, M. Kruskal, T. Kubota, H. Kucuk, S. Kuday, S. Kuehn, A. Kugel, F. Kuger, A. Kuhl, T. Kuhl, V. Kukhtin, Y. Kulchitsky, S. Kuleshov, M. Kuna, T. Kunigo, A. Kupco, H. Kurashige, Y. A. Kurochkin, R. Kurumida, V. Kus, E. S. Kuwertz, M. Kuze, J. Kvita, T. Kwan, D. Kyriazopoulos, A. La Rosa, J. L. La Rosa Navarro, L. La Rotonda, C. Lacasta, F. Lacava, J. Lacey, H. Lacker, D. Lacour, V. R. Lacuesta, E. Ladygin, R. Lafaye, B. Laforge, T. Lagouri, S. Lai, L. Lambourne, S. Lammers, C. L. Lampen, W. Lampl, E. Lançon, U. Landgraf, M. P. J. Landon, V. S. Lang, J. C. Lange, A. J. Lankford, F. Lanni, K. Lantzsch, S. Laplace, C. Lapoire, J. F. Laporte, T. Lari, F. Lasagni Manghi, M. Lassnig, P. Laurelli, W. Lavrijsen, A. T. Law, P. Laycock, O. Le Dortz, E. Le Guirriec, E. Le Menedeu, M. LeBlanc, T. LeCompte, F. Ledroit-Guillon, C. A. Lee, S. C. Lee, L. Lee, G. Lefebvre, M. Lefebvre, F. Legger, C. Leggett, A. Lehan, G. Lehmann Miotto, X. Lei, W. A. Leight, A. Leisos, A. G. Leister, M. A. L. Leite, R. Leitner, D. Lellouch, B. Lemmer, K. J. C. Leney, T. Lenz, B. Lenzi, R. Leone, S. Leone, C. Leonidopoulos, S. Leontsinis, C. Leroy, C. G. Lester, M. Levchenko, J. Levêque, D. Levin, L. J. Levinson, M. Levy, A. Lewis, A. M. Leyko, M. Leyton, B. Li, H. Li, H. L. Li, L. Li, L. Li, S. Li, Y. Li, Z. Liang, H. Liao, B. Liberti, A. Liblong, P. Lichard, K. Lie, J. Liebal, W. Liebig, C. Limbach, A. Limosani, S. C. Lin, T. H. Lin, F. Linde, B. E. Lindquist, J. T. Linnemann, E. Lipeles, A. Lipniacka, M. Lisovyi, T. M. Liss, D. Lissauer, A. Lister, A. M. Litke, B. Liu, D. Liu, J. Liu, J. B. Liu, K. Liu, L. Liu, M. Liu, M. Liu, Y. Liu, M. Livan, A. Lleres, J. Llorente Merino, S. L. Lloyd, F. Lo Sterzo, E. Lobodzinska, P. Loch, W. S. Lockman, F. K. Loebinger, A. E. Loevschall-Jensen, A. Loginov, T. Lohse, K. Lohwasser, M. Lokajicek, B. A. Long, J. D. Long, R. E. Long, K. A. Looper, L. Lopes, D. Lopez Mateos, B. Lopez Paredes, I. Lopez Paz, J. Lorenz, N. Lorenzo Martinez, M. Losada, P. Loscutoff, P. J. Lösel, X. Lou, A. Lounis, J. Love, P. A. Love, N. Lu, H. J. Lubatti, C. Luci, A. Lucotte, F. Luehring, W. Lukas, L. Luminari, O. Lundberg, B. Lund-Jensen, D. Lynn, R. Lysak, E. Lytken, H. Ma, L. L. Ma, G. Maccarrone, A. Macchiolo, C. M. Macdonald, J. Machado Miguens, D. Macina, D. Madaffari, R. Madar, H. J. Maddocks, W. F. Mader, A. Madsen, S. Maeland, T. Maeno, A. Maevskiy, E. Magradze, K. Mahboubi, J. Mahlstedt, C. Maiani, C. Maidantchik, A. A. Maier, T. Maier, A. Maio, S. Majewski, Y. Makida, N. Makovec, B. Malaescu, Pa. Malecki, V. P. Maleev, F. Malek, U. Mallik, D. Malon, C. Malone, S. Maltezos, V. M. Malyshev, S. Malyukov, J. Mamuzic, G. Mancini, B. Mandelli, L. Mandelli, I. Mandić, R. Mandrysch, J. Maneira, A. Manfredini, L. Manhaes de Andrade Filho, J. Manjarres Ramos, A. Mann, P. M. Manning, A. Manousakis-Katsikakis, B. Mansoulie, R. Mantifel, M. Mantoani, L. Mapelli, L. March, G. Marchiori, M. Marcisovsky, C. P. Marino, M. Marjanovic, F. Marroquim, S. P. Marsden, Z. Marshall, L. F. Marti, S. Marti-Garcia, B. Martin, T. A. Martin, V. J. Martin, B. Martin dit Latour, M. Martinez, S. Martin-Haugh, V. S. Martoiu, A. C. Martyniuk, M. Marx, F. Marzano, A. Marzin, L. Masetti, T. Mashimo, R. Mashinistov, J. Masik, A. L. Maslennikov, I. Massa, L. Massa, N. Massol, P. Mastrandrea, A. Mastroberardino, T. Masubuchi, P. Mättig, J. Mattmann, J. Maurer, S. J. Maxfield, D. A. Maximov, R. Mazini, S. M. Mazza, L. Mazzaferro, G. Mc Goldrick, S. P. Mc Kee, A. McCarn, R. L. McCarthy, T. G. McCarthy, N. A. McCubbin, K. W. McFarlane, J. A. Mcfayden, G. Mchedlidze, S. J. McMahon, R. A. McPherson, M. Medinnis, S. Meehan, S. Mehlhase, A. Mehta, K. Meier, C. Meineck, B. Meirose, B. R. Mellado Garcia, F. Meloni, A. Mengarelli, S. Menke, E. Meoni, K. M. Mercurio, S. Mergelmeyer, P. Mermod, L. Merola, C. Meroni, F. S. Merritt, A. Messina, J. Metcalfe, A. S. Mete, C. Meyer, C. Meyer, J-P. Meyer, J. Meyer, R. P. Middleton, S. Miglioranzi, L. Mijović, G. Mikenberg, M. Mikestikova, A. Mikuž, A. Milesi, A. Milic, D. W. Miller, C. Mills, A. Milov, D. A. Milstead, A. A. Minaenko, Y. Minami, I. A. Minashvili, A. I. Mincer, B. Mindur, M. Mineev, Y. Ming, L. M. Mir, T. Mitani, J. Mitrevski, V. A. Mitsou, A. Miucci, P. S. Miyagawa, J. U. Mjörnmark, T. Moa, K. Mochizuki, S. Mohapatra, W. Mohr, S. Molander, R. Moles-Valls, K. Mönig, C. Monini, J. Monk, E. Monnier, J. Montejo Berlingen, F. Monticelli, S. Monzani, R. W. Moore, N. Morange, D. Moreno, M. Moreno Llácer, P. Morettini, M. Morgenstern, M. Morii, M. Morinaga, V. Morisbak, S. Moritz, A. K. Morley, G. Mornacchi, J. D. Morris, S. S. Mortensen, A. Morton, L. Morvaj, H. G. Moser, M. Mosidze, J. Moss, K. Motohashi, R. Mount, E. Mountricha, S. V. Mouraviev, E. J. W. Moyse, S. Muanza, R. D. Mudd, F. Mueller, J. Mueller, K. Mueller, R. S. P. Mueller, T. Mueller, D. Muenstermann, P. Mullen, Y. Munwes, J. A. Murillo Quijada, W. J. Murray, H. Musheghyan, E. Musto, A. G. Myagkov, M. Myska, O. Nackenhorst, J. Nadal, K. Nagai, R. Nagai, Y. Nagai, K. Nagano, A. Nagarkar, Y. Nagasaka, K. Nagata, M. Nagel, E. Nagy, A. M. Nairz, Y. Nakahama, K. Nakamura, T. Nakamura, I. Nakano, H. Namasivayam, R. F. Naranjo Garcia, R. Narayan, T. Naumann, G. Navarro, R. Nayyar, H. A. Neal, P. Yu. Nechaeva, T. J. Neep, P. D. Nef, A. Negri, M. Negrini, S. Nektarijevic, C. Nellist, A. Nelson, S. Nemecek, P. Nemethy, A. A. Nepomuceno, M. Nessi, M. S. Neubauer, M. Neumann, R. M. Neves, P. Nevski, P. R. Newman, D. H. Nguyen, R. B. Nickerson, R. Nicolaidou, B. Nicquevert, J. Nielsen, N. Nikiforou, A. Nikiforov, V. Nikolaenko, I. Nikolic-Audit, K. Nikolopoulos, J. K. Nilsen, P. Nilsson, Y. Ninomiya, A. Nisati, R. Nisius, T. Nobe, M. Nomachi, I. Nomidis, T. Nooney, S. Norberg, M. Nordberg, O. Novgorodova, S. Nowak, M. Nozaki, L. Nozka, K. Ntekas, G. Nunes Hanninger, T. Nunnemann, E. Nurse, F. Nuti, B. J. O’Brien, F. O’grady, D. C. O’Neil, V. O’Shea, F. G. Oakham, H. Oberlack, T. Obermann, J. Ocariz, A. Ochi, I. Ochoa, S. Oda, S. Odaka, H. Ogren, A. Oh, S. H. Oh, C. C. Ohm, H. Ohman, H. Oide, W. Okamura, H. Okawa, Y. Okumura, T. Okuyama, A. Olariu, S. A. Olivares Pino, D. Oliveira Damazio, E. Oliver Garcia, A. Olszewski, J. Olszowska, A. Onofre, P. U. E. Onyisi, C. J. Oram, M. J. Oreglia, Y. Oren, D. Orestano, N. Orlando, C. Oropeza Barrera, R. S. Orr, B. Osculati, R. Ospanov, G. Otero y Garzon, H. Otono, M. Ouchrif, E. A. Ouellette, F. Ould-Saada, A. Ouraou, K. P. Oussoren, Q. Ouyang, A. Ovcharova, M. Owen, R. E. Owen, V. E. Ozcan, N. Ozturk, K. Pachal, A. Pacheco Pages, C. Padilla Aranda, M. Pagáčová, S. Pagan Griso, E. Paganis, C. Pahl, F. Paige, P. Pais, K. Pajchel, G. Palacino, S. Palestini, M. Palka, D. Pallin, A. Palma, Y. B. Pan, E. Panagiotopoulou, C. E. Pandini, J. G. Panduro Vazquez, P. Pani, S. Panitkin, L. Paolozzi, Th. D. Papadopoulou, K. Papageorgiou, A. Paramonov, D. Paredes Hernandez, M. A. Parker, K. A. Parker, F. Parodi, J. A. Parsons, U. Parzefall, E. Pasqualucci, S. Passaggio, F. Pastore, Fr. Pastore, G. Pásztor, S. Pataraia, N. D. Patel, J. R. Pater, T. Pauly, J. Pearce, B. Pearson, L. E. Pedersen, M. Pedersen, S. Pedraza Lopez, R. Pedro, S. V. Peleganchuk, D. Pelikan, H. Peng, B. Penning, J. Penwell, D. V. Perepelitsa, E. Perez Codina, M. T. Pérez García-Estañ, L. Perini, H. Pernegger, S. Perrella, R. Peschke, V. D. Peshekhonov, K. Peters, R. F. Y. Peters, B. A. Petersen, T. C. Petersen, E. Petit, A. Petridis, C. Petridou, E. Petrolo, F. Petrucci, N. E. Pettersson, R. Pezoa, P. W. Phillips, G. Piacquadio, E. Pianori, A. Picazio, E. Piccaro, M. Piccinini, M. A. Pickering, R. Piegaia, D. T. Pignotti, J. E. Pilcher, A. D. Pilkington, J. Pina, M. Pinamonti, J. L. Pinfold, A. Pingel, B. Pinto, S. Pires, M. Pitt, C. Pizio, L. Plazak, M.-A. Pleier, V. Pleskot, E. Plotnikova, P. Plucinski, D. Pluth, R. Poettgen, L. Poggioli, D. Pohl, G. Polesello, A. Policicchio, R. Polifka, A. Polini, C. S. Pollard, V. Polychronakos, K. Pommès, L. Pontecorvo, B. G. Pope, G. A. Popeneciu, D. S. Popovic, A. Poppleton, S. Pospisil, K. Potamianos, I. N. Potrap, C. J. Potter, C. T. Potter, G. Poulard, J. Poveda, V. Pozdnyakov, P. Pralavorio, A. Pranko, S. Prasad, S. Prell, D. Price, L. E. Price, M. Primavera, S. Prince, M. Proissl, K. Prokofiev, F. Prokoshin, E. Protopapadaki, S. Protopopescu, J. Proudfoot, M. Przybycien, E. Ptacek, D. Puddu, E. Pueschel, D. Puldon, M. Purohit, P. Puzo, J. Qian, G. Qin, Y. Qin, A. Quadt, D. R. Quarrie, W. B. Quayle, M. Queitsch-Maitland, D. Quilty, S. Raddum, V. Radeka, V. Radescu, S. K. Radhakrishnan, P. Radloff, P. Rados, F. Ragusa, G. Rahal, S. Rajagopalan, M. Rammensee, C. Rangel-Smith, F. Rauscher, S. Rave, T. Ravenscroft, M. Raymond, A. L. Read, N. P. Readioff, D. M. Rebuzzi, A. Redelbach, G. Redlinger, R. Reece, K. Reeves, L. Rehnisch, H. Reisin, M. Relich, C. Rembser, H. Ren, A. Renaud, M. Rescigno, S. Resconi, O. L. Rezanova, P. Reznicek, R. Rezvani, R. Richter, S. Richter, E. Richter-Was, O. Ricken, M. Ridel, P. Rieck, C. J. Riegel, J. Rieger, M. Rijssenbeek, A. Rimoldi, L. Rinaldi, B. Ristić, E. Ritsch, I. Riu, F. Rizatdinova, E. Rizvi, S. H. Robertson, A. Robichaud-Veronneau, D. Robinson, J. E. M. Robinson, A. Robson, C. Roda, S. Roe, O. Røhne, S. Rolli, A. Romaniouk, M. Romano, S. M. Romano Saez, E. Romero Adam, N. Rompotis, M. Ronzani, L. Roos, E. Ros, S. Rosati, K. Rosbach, P. Rose, P. L. Rosendahl, O. Rosenthal, V. Rossetti, E. Rossi, L. P. Rossi, R. Rosten, M. Rotaru, I. Roth, J. Rothberg, D. Rousseau, C. R. Royon, A. Rozanov, Y. Rozen, X. Ruan, F. Rubbo, I. Rubinskiy, V. I. Rud, C. Rudolph, M. S. Rudolph, F. Rühr, A. Ruiz-Martinez, Z. Rurikova, N. A. Rusakovich, A. Ruschke, H. L. Russell, J. P. Rutherfoord, N. Ruthmann, Y. F. Ryabov, M. Rybar, G. Rybkin, N. C. Ryder, A. F. Saavedra, G. Sabato, S. Sacerdoti, A. Saddique, H. F-W. Sadrozinski, R. Sadykov, F. Safai Tehrani, M. Saimpert, H. Sakamoto, Y. Sakurai, G. Salamanna, A. Salamon, M. Saleem, D. Salek, P. H. Sales De Bruin, D. Salihagic, A. Salnikov, J. Salt, D. Salvatore, F. Salvatore, A. Salvucci, A. Salzburger, D. Sampsonidis, A. Sanchez, J. Sánchez, V. Sanchez Martinez, H. Sandaker, R. L. Sandbach, H. G. Sander, M. P. Sanders, M. Sandhoff, C. Sandoval, R. Sandstroem, D. P. C. Sankey, M. Sannino, A. Sansoni, C. Santoni, R. Santonico, H. Santos, I. Santoyo Castillo, K. Sapp, A. Sapronov, J. G. Saraiva, B. Sarrazin, O. Sasaki, Y. Sasaki, K. Sato, G. Sauvage, E. Sauvan, G. Savage, P. Savard, C. Sawyer, L. Sawyer, J. Saxon, C. Sbarra, A. Sbrizzi, T. Scanlon, D. A. Scannicchio, M. Scarcella, V. Scarfone, J. Schaarschmidt, P. Schacht, D. Schaefer, R. Schaefer, J. Schaeffer, S. Schaepe, S. Schaetzel, U. Schäfer, A. C. Schaffer, D. Schaile, R. D. Schamberger, V. Scharf, V. A. Schegelsky, D. Scheirich, M. Schernau, C. Schiavi, C. Schillo, M. Schioppa, S. Schlenker, E. Schmidt, K. Schmieden, C. Schmitt, S. Schmitt, S. Schmitt, B. Schneider, Y. J. Schnellbach, U. Schnoor, L. Schoeffel, A. Schoening, B. D. Schoenrock, E. Schopf, A. L. S. Schorlemmer, M. Schott, D. Schouten, J. Schovancova, S. Schramm, M. Schreyer, C. Schroeder, N. Schuh, M. J. Schultens, H.-C. Schultz-Coulon, H. Schulz, M. Schumacher, B. A. Schumm, Ph. Schune, C. Schwanenberger, A. Schwartzman, T. A. Schwarz, Ph. Schwegler, Ph. Schwemling, R. Schwienhorst, J. Schwindling, T. Schwindt, M. Schwoerer, F. G. Sciacca, E. Scifo, G. Sciolla, F. Scuri, F. Scutti, J. Searcy, G. Sedov, E. Sedykh, P. Seema, S. C. Seidel, A. Seiden, F. Seifert, J. M. Seixas, G. Sekhniaidze, K. Sekhon, S. J. Sekula, K. E. Selbach, D. M. Seliverstov, N. Semprini-Cesari, C. Serfon, L. Serin, L. Serkin, T. Serre, M. Sessa, R. Seuster, H. Severini, T. Sfiligoj, F. Sforza, A. Sfyrla, E. Shabalina, M. Shamim, L. Y. Shan, R. Shang, J. T. Shank, M. Shapiro, P. B. Shatalov, K. Shaw, S. M Shaw, A. Shcherbakova, C. Y. Shehu, P. Sherwood, L. Shi, S. Shimizu, C. O. Shimmin, M. Shimojima, M. Shiyakova, A. Shmeleva, D. Shoaleh Saadi, M. J. Shochet, S. Shojaii, S. Shrestha, E. Shulga, M. A. Shupe, S. Shushkevich, P. Sicho, O. Sidiropoulou, D. Sidorov, A. Sidoti, F. Siegert, Dj. Sijacki, J. Silva, Y. Silver, S. B. Silverstein, V. Simak, O. Simard, Lj. Simic, S. Simion, E. Simioni, B. Simmons, D. Simon, R. Simoniello, P. Sinervo, N. B. Sinev, G. Siragusa, A. N. Sisakyan, S. Yu. Sivoklokov, J. Sjölin, T. B. Sjursen, M. B. Skinner, H. P. Skottowe, P. Skubic, M. Slater, T. Slavicek, M. Slawinska, K. Sliwa, V. Smakhtin, B. H. Smart, L. Smestad, S. Yu. Smirnov, Y. Smirnov, L. N. Smirnova, O. Smirnova, M. N. K. Smith, M. Smizanska, K. Smolek, A. A. Snesarev, G. Snidero, S. Snyder, R. Sobie, F. Socher, A. Soffer, D. A. Soh, C. A. Solans, M. Solar, J. Solc, E. Yu. Soldatov, U. Soldevila, A. A. Solodkov, A. Soloshenko, O. V. Solovyanov, V. Solovyev, P. Sommer, H. Y. Song, N. Soni, A. Sood, A. Sopczak, B. Sopko, V. Sopko, V. Sorin, D. Sosa, M. Sosebee, C. L. Sotiropoulou, R. Soualah, P. Soueid, A. M. Soukharev, D. South, S. Spagnolo, M. Spalla, F. Spanò, W. R. Spearman, F. Spettel, R. Spighi, G. Spigo, L. A. Spiller, M. Spousta, T. Spreitzer, R. D. St. Denis, S. Staerz, J. Stahlman, R. Stamen, S. Stamm, E. Stanecka, C. Stanescu, M. Stanescu-Bellu, M. M. Stanitzki, S. Stapnes, E. A. Starchenko, J. Stark, P. Staroba, P. Starovoitov, R. Staszewski, P. Stavina, P. Steinberg, B. Stelzer, H. J. Stelzer, O. Stelzer-Chilton, H. Stenzel, S. Stern, G. A. Stewart, J. A. Stillings, M. C. Stockton, M. Stoebe, G. Stoicea, P. Stolte, S. Stonjek, A. R. Stradling, A. Straessner, M. E. Stramaglia, J. Strandberg, S. Strandberg, A. Strandlie, E. Strauss, M. Strauss, P. Strizenec, R. Ströhmer, D. M. Strom, R. Stroynowski, A. Strubig, S. A. Stucci, B. Stugu, N. A. Styles, D. Su, J. Su, R. Subramaniam, A. Succurro, Y. Sugaya, C. Suhr, M. Suk, V. V. Sulin, S. Sultansoy, T. Sumida, S. Sun, X. Sun, J. E. Sundermann, K. Suruliz, G. Susinno, M. R. Sutton, S. Suzuki, Y. Suzuki, M. Svatos, S. Swedish, M. Swiatlowski, I. Sykora, T. Sykora, D. Ta, C. Taccini, K. Tackmann, J. Taenzer, A. Taffard, R. Tafirout, N. Taiblum, H. Takai, R. Takashima, H. Takeda, T. Takeshita, Y. Takubo, M. Talby, A. A. Talyshev, J. Y. C. Tam, K. G. Tan, J. Tanaka, R. Tanaka, S. Tanaka, B. B. Tannenwald, N. Tannoury, S. Tapprogge, S. Tarem, F. Tarrade, G. F. Tartarelli, P. Tas, M. Tasevsky, T. Tashiro, E. Tassi, A. Tavares Delgado, Y. Tayalati, F. E. Taylor, G. N. Taylor, W. Taylor, F. A. Teischinger, M. Teixeira Dias Castanheira, P. Teixeira-Dias, K. K. Temming, H. Ten Kate, P. K. Teng, J. J. Teoh, F. Tepel, S. Terada, K. Terashi, J. Terron, S. Terzo, M. Testa, R. J. Teuscher, J. Therhaag, T. Theveneaux-Pelzer, J. P. Thomas, J. Thomas-Wilsker, E. N. Thompson, P. D. Thompson, R. J. Thompson, A. S. Thompson, L. A. Thomsen, E. Thomson, M. Thomson, R. P. Thun, M. J. Tibbetts, R. E. Ticse Torres, V. O. Tikhomirov, Yu. A. Tikhonov, S. Timoshenko, E. Tiouchichine, P. Tipton, S. Tisserant, T. Todorov, S. Todorova-Nova, J. Tojo, S. Tokár, K. Tokushuku, K. Tollefson, E. Tolley, L. Tomlinson, M. Tomoto, L. Tompkins, K. Toms, E. Torrence, H. Torres, E. Torró Pastor, J. Toth, F. Touchard, D. R. Tovey, T. Trefzger, L. Tremblet, A. Tricoli, I. M. Trigger, S. Trincaz-Duvoid, M. F. Tripiana, W. Trischuk, B. Trocmé, C. Troncon, M. Trottier-McDonald, M. Trovatelli, P. True, L. Truong, M. Trzebinski, A. Trzupek, C. Tsarouchas, J. C-L. Tseng, P. V. Tsiareshka, D. Tsionou, G. Tsipolitis, N. Tsirintanis, S. Tsiskaridze, V. Tsiskaridze, E. G. Tskhadadze, I. I. Tsukerman, V. Tsulaia, S. Tsuno, D. Tsybychev, A. Tudorache, V. Tudorache, A. N. Tuna, S. A. Tupputi, S. Turchikhin, D. Turecek, R. Turra, A. J. Turvey, P. M. Tuts, A. Tykhonov, M. Tylmad, M. Tyndel, I. Ueda, R. Ueno, M. Ughetto, M. Ugland, M. Uhlenbrock, F. Ukegawa, G. Unal, A. Undrus, G. Unel, F. C. Ungaro, Y. Unno, C. Unverdorben, J. Urban, P. Urquijo, P. Urrejola, G. Usai, A. Usanova, L. Vacavant, V. Vacek, B. Vachon, C. Valderanis, N. Valencic, S. Valentinetti, A. Valero, L. Valery, S. Valkar, E. Valladolid Gallego, S. Vallecorsa, J. A. Valls Ferrer, W. Van Den Wollenberg, P. C. Van Der Deijl, R. van der Geer, H. van der Graaf, R. Van Der Leeuw, N. van Eldik, P. van Gemmeren, J. Van Nieuwkoop, I. van Vulpen, M. C. van Woerden, M. Vanadia, W. Vandelli, R. Vanguri, A. Vaniachine, F. Vannucci, G. Vardanyan, R. Vari, E. W. Varnes, T. Varol, D. Varouchas, A. Vartapetian, K. E. Varvell, F. Vazeille, T. Vazquez Schroeder, J. Veatch, F. Veloso, T. Velz, S. Veneziano, A. Ventura, D. Ventura, M. Venturi, N. Venturi, A. Venturini, V. Vercesi, M. Verducci, W. Verkerke, J. C. Vermeulen, A. Vest, M. C. Vetterli, O. Viazlo, I. Vichou, T. Vickey, O. E. Vickey Boeriu, G. H. A. Viehhauser, S. Viel, R. Vigne, M. Villa, M. Villaplana Perez, E. Vilucchi, M. G. Vincter, V. B. Vinogradov, I. Vivarelli, F. Vives Vaque, S. Vlachos, D. Vladoiu, M. Vlasak, M. Vogel, P. Vokac, G. Volpi, M. Volpi, H. von der Schmitt, H. von Radziewski, E. von Toerne, V. Vorobel, K. Vorobev, M. Vos, R. Voss, J. H. Vossebeld, N. Vranjes, M. Vranjes Milosavljevic, V. Vrba, M. Vreeswijk, R. Vuillermet, I. Vukotic, Z. Vykydal, P. Wagner, W. Wagner, H. Wahlberg, S. Wahrmund, J. Wakabayashi, J. Walder, R. Walker, W. Walkowiak, C. Wang, F. Wang, H. Wang, H. Wang, J. Wang, J. Wang, K. Wang, R. Wang, S. M. Wang, T. Wang, X. Wang, C. Wanotayaroj, A. Warburton, C. P. Ward, D. R. Wardrope, M. Warsinsky, A. Washbrook, C. Wasicki, P. M. Watkins, A. T. Watson, I. J. Watson, M. F. Watson, G. Watts, S. Watts, B. M. Waugh, S. Webb, M. S. Weber, S. W. Weber, J. S. Webster, A. R. Weidberg, B. Weinert, J. Weingarten, C. Weiser, H. Weits, P. S. Wells, T. Wenaus, T. Wengler, S. Wenig, N. Wermes, M. Werner, P. Werner, M. Wessels, J. Wetter, K. Whalen, A. M. Wharton, A. White, M. J. White, R. White, S. White, D. Whiteson, F. J. Wickens, W. Wiedenmann, M. Wielers, P. Wienemann, C. Wiglesworth, L. A. M. Wiik-Fuchs, A. Wildauer, H. G. Wilkens, H. H. Williams, S. Williams, C. Willis, S. Willocq, A. Wilson, J. A. Wilson, I. Wingerter-Seez, F. Winklmeier, B. T. Winter, M. Wittgen, J. Wittkowski, S. J. Wollstadt, M. W. Wolter, H. Wolters, B. K. Wosiek, J. Wotschack, M. J. Woudstra, K. W. Wozniak, M. Wu, M. Wu, S. L. Wu, X. Wu, Y. Wu, T. R. Wyatt, B. M. Wynne, S. Xella, D. Xu, L. Xu, B. Yabsley, S. Yacoob, R. Yakabe, M. Yamada, Y. Yamaguchi, A. Yamamoto, S. Yamamoto, T. Yamanaka, K. Yamauchi, Y. Yamazaki, Z. Yan, H. Yang, H. Yang, Y. Yang, L. Yao, W-M. Yao, Y. Yasu, E. Yatsenko, K. H. Yau Wong, J. Ye, S. Ye, I. Yeletskikh, A. L. Yen, E. Yildirim, K. Yorita, R. Yoshida, K. Yoshihara, C. Young, C. J. S. Young, S. Youssef, D. R. Yu, J. Yu, J. M. Yu, J. Yu, L. Yuan, A. Yurkewicz, I. Yusuff, B. Zabinski, R. Zaidan, A. M. Zaitsev, J. Zalieckas, A. Zaman, S. Zambito, L. Zanello, D. Zanzi, C. Zeitnitz, M. Zeman, A. Zemla, K. Zengel, O. Zenin, T. Ženiš, D. Zerwas, D. Zhang, F. Zhang, J. Zhang, L. Zhang, R. Zhang, X. Zhang, Z. Zhang, X. Zhao, Y. Zhao, Z. Zhao, A. Zhemchugov, J. Zhong, B. Zhou, C. Zhou, L. Zhou, L. Zhou, N. Zhou, C. G. Zhu, H. Zhu, J. Zhu, Y. Zhu, X. Zhuang, K. Zhukov, A. Zibell, D. Zieminska, N. I. Zimine, C. Zimmermann, S. Zimmermann, Z. Zinonos, M. Zinser, M. Ziolkowski, L. Živković, G. Zobernig, A. Zoccoli, M. zur Nedden, G. Zurzolo, L. Zwalinski, ATLAS collaboration

**Affiliations:** Department of Physics, University of Adelaide, Adelaide, Australia; Physics Department, SUNY Albany, Albany, NY USA; Department of Physics, University of Alberta, Edmonton, AB Canada; Department of Physics, Ankara University, Ankara, Turkey; Istanbul Aydin University, Istanbul, Turkey; Division of Physics, TOBB University of Economics and Technology, Ankara, Turkey; LAPP, CNRS/IN2P3 and Université Savoie Mont Blanc, Annecy-le-Vieux, France; High Energy Physics Division, Argonne National Laboratory, Argonne, IL USA; Department of Physics, University of Arizona, Tucson, AZ USA; Department of Physics, The University of Texas at Arlington, Arlington, TX USA; Physics Department, University of Athens, Athens, Greece; Physics Department, National Technical University of Athens, Zografou, Greece; Institute of Physics, Azerbaijan Academy of Sciences, Baku, Azerbaijan; Institut de Física d’Altes Energies and Departament de Física de la Universitat Autònoma de Barcelona, Barcelona, Spain; Institute of Physics, University of Belgrade, Belgrade, Serbia; Department for Physics and Technology, University of Bergen, Bergen, Norway; Physics Division, Lawrence Berkeley National Laboratory and University of California, Berkeley, CA USA; Department of Physics, Humboldt University, Berlin, Germany; Albert Einstein Center for Fundamental Physics and Laboratory for High Energy Physics, University of Bern, Bern, Switzerland; School of Physics and Astronomy, University of Birmingham, Birmingham, UK; Department of Physics, Bogazici University, Istanbul, Turkey; Department of Physics, Dogus University, Istanbul, Turkey; Department of Physics Engineering, Gaziantep University, Gaziantep, Turkey; INFN Sezione di Bologna, Bologna, Italy; Dipartimento di Fisica e Astronomia, Università di Bologna, Bologna, Italy; Physikalisches Institut, University of Bonn, Bonn, Germany; Department of Physics, Boston University, Boston, MA USA; Department of Physics, Brandeis University, Waltham, MA USA; Universidade Federal do Rio De Janeiro COPPE/EE/IF, Rio de Janeiro, Brazil; Electrical Circuits Department, Federal University of Juiz de Fora (UFJF), Juiz de Fora, Brazil; Federal University of Sao Joao del Rei (UFSJ), Sao Joao del Rei, Brazil; Instituto de Fisica, Universidade de Sao Paulo, São Paulo, Brazil; Physics Department, Brookhaven National Laboratory, Upton, NY USA; National Institute of Physics and Nuclear Engineering, Bucharest, Romania; Physics Department, National Institute for Research and Development of Isotopic and Molecular Technologies, Cluj Napoca, Romania; University Politehnica Bucharest, Bucharest, Romania; West University in Timisoara, Timisoara, Romania; Departamento de Física, Universidad de Buenos Aires, Buenos Aires, Argentina; Cavendish Laboratory, University of Cambridge, Cambridge, UK; Department of Physics, Carleton University, Ottawa, ON Canada; CERN, Geneva, Switzerland; Enrico Fermi Institute, University of Chicago, Chicago, IL USA; Departamento de Física, Pontificia Universidad Católica de Chile, Santiago, Chile; Departamento de Física, Universidad Técnica Federico Santa María, Valparaiso, Chile; Institute of High Energy Physics, Chinese Academy of Sciences, Beijing, China; Department of Modern Physics, University of Science and Technology of China, Anhui, China; Department of Physics, Nanjing University, Jiangsu, China; School of Physics, Shandong University, Shandong, China; Department of Physics and Astronomy, Shanghai Key Laboratory for Particle Physics and Cosmology, Shanghai Jiao Tong University, Shanghai, China; Physics Department, Tsinghua University, Beijing, 100084 China; Laboratoire de Physique Corpusculaire, Clermont Université and Université Blaise Pascal and CNRS/IN2P3, Clermont-Ferrand, France; Nevis Laboratory, Columbia University, Irvington, NY USA; Niels Bohr Institute, University of Copenhagen, Copenhagen, Denmark; INFN Gruppo Collegato di Cosenza, Laboratori Nazionali di Frascati, Frascati, Italy; Dipartimento di Fisica, Università della Calabria, Rende, Italy; AGH University of Science and Technology, Faculty of Physics and Applied Computer Science, Kraków, Poland; Marian Smoluchowski Institute of Physics, Jagiellonian University, Kraków, Poland; Institute of Nuclear Physics, Polish Academy of Sciences, Kraków, Poland; Physics Department, Southern Methodist University, Dallas, TX USA; Physics Department, University of Texas at Dallas, Richardson, TX USA; DESY, Hamburg and Zeuthen, Germany; Institut für Experimentelle Physik IV, Technische Universität Dortmund, Dortmund, Germany; Institut für Kern- und Teilchenphysik, Technische Universität Dresden, Dresden, Germany; Department of Physics, Duke University, Durham, NC USA; SUPA-School of Physics and Astronomy, University of Edinburgh, Edinburgh, UK; INFN Laboratori Nazionali di Frascati, Frascati, Italy; Fakultät für Mathematik und Physik, Albert-Ludwigs-Universität, Freiburg, Germany; Section de Physique, Université de Genève, Geneva, Switzerland; INFN Sezione di Genova, Genova, Italy; Dipartimento di Fisica, Università di Genova, Genoa, Italy; E. Andronikashvili Institute of Physics, Iv. Javakhishvili Tbilisi State University, Tbilisi, Georgia; High Energy Physics Institute, Tbilisi State University, Tbilisi, Georgia; II Physikalisches Institut, Justus-Liebig-Universität Giessen, Giessen, Germany; SUPA-School of Physics and Astronomy, University of Glasgow, Glasgow, UK; II Physikalisches Institut, Georg-August-Universität, Göttingen, Germany; Laboratoire de Physique Subatomique et de Cosmologie, Université Grenoble-Alpes, CNRS/IN2P3, Grenoble, France; Department of Physics, Hampton University, Hampton, VA USA; Laboratory for Particle Physics and Cosmology, Harvard University, Cambridge, MA USA; Kirchhoff-Institut für Physik, Ruprecht-Karls-Universität Heidelberg, Heidelberg, Germany; Physikalisches Institut, Ruprecht-Karls-Universität Heidelberg, Heidelberg, Germany; ZITI Institut für technische Informatik, Ruprecht-Karls-Universität Heidelberg, Mannheim, Germany; Faculty of Applied Information Science, Hiroshima Institute of Technology, Hiroshima, Japan; Department of Physics, The Chinese University of Hong Kong, Shatin, NT, Hong Kong; Department of Physics, The University of Hong Kong, Pok Fu Lam, Hong Kong; Department of Physics, The Hong Kong University of Science and Technology, Clear Water Bay, Kowloon, Hong Kong, China; Department of Physics, Indiana University, Bloomington, IN USA; Institut für Astro- und Teilchenphysik, Leopold-Franzens-Universität, Innsbruck, Austria; University of Iowa, Iowa City, IA USA; Department of Physics and Astronomy, Iowa State University, Ames, IA USA; Joint Institute for Nuclear Research, JINR Dubna, Dubna, Russia; KEK, High Energy Accelerator Research Organization, Tsukuba, Japan; Graduate School of Science, Kobe University, Kobe, Japan; Faculty of Science, Kyoto University, Kyoto, Japan; Kyoto University of Education, Kyoto, Japan; Department of Physics, Kyushu University, Fukuoka, Japan; Instituto de Física La Plata, Universidad Nacional de La Plata and CONICET, La Plata, Argentina; Physics Department, Lancaster University, Lancaster, UK; INFN Sezione di Lecce, Lecce, Italy; Dipartimento di Matematica e Fisica, Università del Salento, Lecce, Italy; Oliver Lodge Laboratory, University of Liverpool, Liverpool, UK; Department of Physics, Jožef Stefan Institute and University of Ljubljana, Ljubljana, Slovenia; School of Physics and Astronomy, Queen Mary University of London, London, UK; Department of Physics, Royal Holloway University of London, Surrey, UK; Department of Physics and Astronomy, University College London, London, UK; Louisiana Tech University, Ruston, LA USA; Laboratoire de Physique Nucléaire et de Hautes Energies, UPMC and Université Paris-Diderot and CNRS/IN2P3, Paris, France; Fysiska institutionen, Lunds universitet, Lund, Sweden; Departamento de Fisica Teorica C-15, Universidad Autonoma de Madrid, Madrid, Spain; Institut für Physik, Universität Mainz, Mainz, Germany; School of Physics and Astronomy, University of Manchester, Manchester, UK; CPPM, Aix-Marseille Université and CNRS/IN2P3, Marseille, France; Department of Physics, University of Massachusetts, Amherst, MA USA; Department of Physics, McGill University, Montreal, QC Canada; School of Physics, University of Melbourne, Melbourne, VIC Australia; Department of Physics, The University of Michigan, Ann Arbor, MI USA; Department of Physics and Astronomy, Michigan State University, East Lansing, MI USA; INFN Sezione di Milano, Milan, Italy; Dipartimento di Fisica, Università di Milano, Milan, Italy; B.I. Stepanov Institute of Physics, National Academy of Sciences of Belarus, Minsk, Republic of Belarus; National Scientific and Educational Centre for Particle and High Energy Physics, Minsk, Republic of Belarus; Department of Physics, Massachusetts Institute of Technology, Cambridge, MA USA; Group of Particle Physics, University of Montreal, Montreal, QC Canada; P.N. Lebedev Institute of Physics, Academy of Sciences, Moscow, Russia; Institute for Theoretical and Experimental Physics (ITEP), Moscow, Russia; National Research Nuclear University MEPhI, Moscow, Russia; D.V. Skobeltsyn Institute of Nuclear Physics, M.V. Lomonosov Moscow State University, Moscow, Russia; Fakultät für Physik, Ludwig-Maximilians-Universität München, Munich, Germany; Max-Planck-Institut für Physik (Werner-Heisenberg-Institut), Munich, Germany; Nagasaki Institute of Applied Science, Nagasaki, Japan; Graduate School of Science and Kobayashi-Maskawa Institute, Nagoya University, Nagoya, Japan; INFN Sezione di Napoli, Naples, Italy; Dipartimento di Fisica, Università di Napoli, Naples, Italy; Department of Physics and Astronomy, University of New Mexico, Albuquerque, NM USA; Institute for Mathematics, Astrophysics and Particle Physics, Radboud University Nijmegen/Nikhef, Nijmegen, The Netherlands; Nikhef National Institute for Subatomic Physics and University of Amsterdam, Amsterdam, The Netherlands; Department of Physics, Northern Illinois University, De Kalb, IL USA; Budker Institute of Nuclear Physics, SB RAS, Novosibirsk, Russia; Department of Physics, New York University, New York, NY USA; Ohio State University, Columbus, OH USA; Faculty of Science, Okayama University, Okayama, Japan; Homer L. Dodge Department of Physics and Astronomy, University of Oklahoma, Norman, OK USA; Department of Physics, Oklahoma State University, Stillwater, OK USA; Palacký University, RCPTM, Olomouc, Czech Republic; Center for High Energy Physics, University of Oregon, Eugene, OR USA; LAL, Université Paris-Sud and CNRS/IN2P3, Orsay, France; Graduate School of Science, Osaka University, Osaka, Japan; Department of Physics, University of Oslo, Oslo, Norway; Department of Physics, Oxford University, Oxford, UK; INFN Sezione di Pavia, Pavia, Italy; Dipartimento di Fisica, Università di Pavia, Pavia, Italy; Department of Physics, University of Pennsylvania, Philadelphia, PA USA; Petersburg Nuclear Physics Institute, Gatchina, Russia; INFN Sezione di Pisa, Pisa, Italy; Dipartimento di Fisica E. Fermi, Università di Pisa, Pisa, Italy; Department of Physics and Astronomy, University of Pittsburgh, Pittsburgh, PA USA; Laboratorio de Instrumentacao e Fisica Experimental de Particulas-LIP, Lisbon, Portugal; Faculdade de Ciências, Universidade de Lisboa, Lisbon, Portugal; Department of Physics, University of Coimbra, Coimbra, Portugal; Centro de Física Nuclear da Universidade de Lisboa, Lisbon, Portugal; Departamento de Fisica, Universidade do Minho, Braga, Portugal; Departamento de Fisica Teorica y del Cosmos and CAFPE, Universidad de Granada, Granada, Spain; Dep Fisica and CEFITEC of Faculdade de Ciencias e Tecnologia, Universidade Nova de Lisboa, Caparica, Portugal; Institute of Physics, Academy of Sciences of the Czech Republic, Prague, Czech Republic; Czech Technical University in Prague, Prague, Czech Republic; Faculty of Mathematics and Physics, Charles University in Prague, Prague, Czech Republic; State Research Center Institute for High Energy Physics, Protvino, Russia; Particle Physics Department, Rutherford Appleton Laboratory, Didcot, UK; INFN Sezione di Roma, Rome, Italy; Dipartimento di Fisica, Sapienza Università di Roma, Rome, Italy; INFN Sezione di Roma Tor Vergata, Rome, Italy; Dipartimento di Fisica, Università di Roma Tor Vergata, Rome, Italy; INFN Sezione di Roma Tre, Rome, Italy; Dipartimento di Matematica e Fisica, Università Roma Tre, Rome, Italy; Faculté des Sciences Ain Chock, Réseau Universitaire de Physique des Hautes Energies-Université Hassan II, Casablanca, Morocco; Centre National de l’Energie des Sciences Techniques Nucleaires, Rabat, Morocco; Faculté des Sciences Semlalia, Université Cadi Ayyad, LPHEA-Marrakech, Marrakech, Morocco; Faculté des Sciences, Université Mohamed Premier and LPTPM, Oujda, Morocco; Faculté des Sciences, Université Mohammed V-Agdal, Rabat, Morocco; DSM/IRFU (Institut de Recherches sur les Lois Fondamentales de l’Univers), CEA Saclay (Commissariat à l’Energie Atomique et aux Energies Alternatives), Gif-sur-Yvette, France; Santa Cruz Institute for Particle Physics, University of California Santa Cruz, Santa Cruz, CA USA; Department of Physics, University of Washington, Seattle, WA USA; Department of Physics and Astronomy, University of Sheffield, Sheffield, UK; Department of Physics, Shinshu University, Nagano, Japan; Fachbereich Physik, Universität Siegen, Siegen, Germany; Department of Physics, Simon Fraser University, Burnaby, BC Canada; SLAC National Accelerator Laboratory, Stanford, CA USA; Faculty of Mathematics, Physics and Informatics, Comenius University, Bratislava, Slovak Republic; Department of Subnuclear Physics, Institute of Experimental Physics of the Slovak Academy of Sciences, Kosice, Slovak Republic; Department of Physics, University of Cape Town, Cape Town, South Africa; Department of Physics, University of Johannesburg, Johannesburg, South Africa; School of Physics, University of the Witwatersrand, Johannesburg, South Africa; Department of Physics, Stockholm University, Stockholm, Sweden; The Oskar Klein Centre, Stockholm, Sweden; Physics Department, Royal Institute of Technology, Stockholm, Sweden; Departments of Physics and Astronomy and Chemistry, Stony Brook University, Stony Brook, NY USA; Department of Physics and Astronomy, University of Sussex, Brighton, UK; School of Physics, University of Sydney, Sydney, Australia; Institute of Physics, Academia Sinica, Taipei, Taiwan; Department of Physics, Technion: Israel Institute of Technology, Haifa, Israel; Raymond and Beverly Sackler School of Physics and Astronomy, Tel Aviv University, Tel Aviv, Israel; Department of Physics, Aristotle University of Thessaloniki, Thessaloníki, Greece; International Center for Elementary Particle Physics and Department of Physics, The University of Tokyo, Tokyo, Japan; Graduate School of Science and Technology, Tokyo Metropolitan University, Tokyo, Japan; Department of Physics, Tokyo Institute of Technology, Tokyo, Japan; Department of Physics, University of Toronto, Toronto, ON Canada; TRIUMF, Vancouver, BC, Canada; Department of Physics and Astronomy, York University, Toronto, ON Canada; Faculty of Pure and Applied Sciences, University of Tsukuba, Tsukuba, Japan; Department of Physics and Astronomy, Tufts University, Medford, MA USA; Centro de Investigaciones, Universidad Antonio Narino, Bogotá, Colombia; Department of Physics and Astronomy, University of California Irvine, Irvine, CA USA; INFN Gruppo Collegato di Udine, Sezione di Trieste, Udine, Italy; ICTP, Trieste, Italy; Dipartimento di Chimica, Fisica e Ambiente, Università di Udine, Udine, Italy; Department of Physics, University of Illinois, Urbana, IL USA; Department of Physics and Astronomy, University of Uppsala, Uppsala, Sweden; Instituto de Física Corpuscular (IFIC) and Departamento de Física Atómica, Molecular y Nuclear and Departamento de Ingeniería Electrónica and Instituto de Microelectrónica de Barcelona (IMB-CNM), University of Valencia and CSIC, Valencia, Spain; Department of Physics, University of British Columbia, Vancouver, BC Canada; Department of Physics and Astronomy, University of Victoria, Victoria, BC Canada; Department of Physics, University of Warwick, Coventry, UK; Waseda University, Tokyo, Japan; Department of Particle Physics, The Weizmann Institute of Science, Rehovot, Israel; Department of Physics, University of Wisconsin, Madison, WI USA; Fakultät für Physik und Astronomie, Julius-Maximilians-Universität, Würzburg, Germany; Fachbereich C Physik, Bergische Universität Wuppertal, Wuppertal, Germany; Department of Physics, Yale University, New Haven, CT USA; Yerevan Physics Institute, Yerevan, Armenia; Centre de Calcul de l’Institut National de Physique Nucléaire et de Physique des Particules (IN2P3), Villeurbanne, France; CERN, Geneva, Switzerland

## Abstract

A search for Higgs boson decays to invisible particles is performed using 20.3 ${{\mathrm {fb}}^{-1}}$ of *pp* collision data at a centre-of-mass energy of 8 TeV recorded by the ATLAS detector at the Large Hadron Collider. The process considered is Higgs boson production in association with a vector boson ($V = W$ or *Z*) that decays hadronically, resulting in events with two or more jets and large missing transverse momentum. No excess of candidates is observed in the data over the background expectation. The results are used to constrain *VH* production followed by *H* decaying to invisible particles for the Higgs boson mass range $115<m_H<300$ GeV. The 95 % confidence-level observed upper limit on $\sigma _{VH} \times \text {BR}(H\rightarrow \text {inv.})$ varies from 1.6 pb at 115 GeV to 0.13 pb at 300 GeV. Assuming Standard Model production and including the $gg\rightarrow H$ contribution as signal, the results also lead to an observed upper limit of 78 % at 95 % confidence level on the branching ratio of Higgs bosons decays to invisible particles at a mass of 125 GeV.

## Introduction

Since the discovery of a Higgs boson with a mass of approximately 125 GeV [1, 2] at the LHC in 2012, the properties of this new particle have been studied extensively. All results obtained so far [3–9] are consistent with the expectations of the long-sought Standard Model (SM) Higgs boson [10–13]. However, sizeable deviations from the SM expectation cannot be yet excluded; the total branching ratio of beyond-the-SM decays of the Higgs boson is only weakly constrained, and its value could be as high as $\sim$40 % [8, 14]. One possible decay is to weakly interacting particles, as predicted by many extensions of the SM, e.g. Higgs boson portal models [15–18]. In these models, the Higgs boson can decay to a pair of dark-matter particles if kinematically allowed. These decays are generally “invisible” to detectors, resulting in events with large missing transverse momentum (${E_{\mathrm{T}}^{\mathrm{miss}}}$).

Searches for Higgs boson decays to invisible particles ($H\rightarrow \text {inv.}$) have been performed by both the ATLAS and CMS collaborations [14, 19]. For example, the ATLAS Collaboration has placed an upper limit of 75 % [19] on the branching ratio of $H\rightarrow \text {inv.}$ from Higgs boson production in association with a *Z* boson identified from its leptonic decays ($Z\rightarrow ee,\mu \mu$). The present paper describes an independent search for the $H\rightarrow \text {inv.}$ decay in final states with two or more jets and large ${E_{\mathrm{T}}^{\mathrm{miss}}}$, motivated by Higgs boson production in association with a vector boson *V* ($V=W$ or *Z*): $q\bar{q}'\rightarrow VH$. The vector boson is identified through its decay to a pair of quarks, reconstructed as hadronic jets in the ATLAS detector, $V\rightarrow jj$. Gluon fusion production $gg\rightarrow H$ followed by $H\rightarrow \text {inv.}$ can also lead to events with two or more jets and large ${E_{\mathrm{T}}^{\mathrm{miss}}}$, and therefore contributes to the signal of the search. Negligible contributions of approximately 1 and 0.2 % to the sensitivity come from $q\bar{q}'\rightarrow q\bar{q}'H$ production via vector-boson fusion (VBF) and from $qq/gg\rightarrow t\bar{t}H$ (*ttH*) production, respectively. The VBF contribution is strongly suppressed by the $m_{jj}$ (dijet invariant mass) window cuts and by the forward-jet veto used to reduce the top quark-antiquark background ($t\bar{t}$), as described in Sect. 4. In a previous ATLAS dark-matter search, limits on Higgs boson decays to invisible particles in *VH* production were set using events with a hadronically decaying vector boson and ${E_{\mathrm{T}}^{\mathrm{miss}}}$ as well [20]. However, the present analysis achieves better sensitivity by using different techniques and performing dedicated optimizations.

## Experimental setup

This search is based on proton–proton collision data at a centre-of-mass energy of 8 TeV recorded with the ATLAS detector [21] in 2012, corresponding to an integrated luminosity of 20.3 ${{\mathrm {fb}}^{-1}}$. The ATLAS detector is a general-purpose detector with an inner tracking system, electromagnetic and hadronic calorimeters, and a muon spectrometer surrounding the interaction point.^1^ The inner tracking system is immersed in a 2 T axial magnetic field, and the muon spectrometer employs a toroidal magnetic field. Only data recorded when all subdetector systems were functional are used in this analysis.

The trigger system is organised in three levels. The first level is based on custom-made hardware and uses coarse-granularity calorimeter and muon information. The second and third levels are implemented as software algorithms and use the full detector granularity. At the second level, only regions deemed interesting at the first level are analysed, while the third level, called the event filter, makes use of the full detector read-out to reconstruct and select events, which are then logged for offline analysis at a rate of up to 400 Hz averaged over an accelerator fill.

## Object reconstruction and simulated samples

Jets are reconstructed using the anti-$k_t$ algorithm [22] with a radius parameter of $R=0.4$. Jet energies are corrected for the average contributions from minimum-bias interactions within the same bunch crossing as the hard-scattering process and within neighbouring bunch crossings (pile-up). Furthermore, for jets with ${p_{\mathrm{T}}}<50$ GeV and $|\eta |<2.4$, the scalar sum of the ${p_{\mathrm{T}}}$ of tracks matched to the jet and originating from the primary vertex^2^ must be at least 50 % of the scalar sum of the ${p_{\mathrm{T}}}$ of all tracks matched to the jet, to suppress jets from pile-up interactions. Jets must have ${p_{\mathrm{T}}}>20$ GeV (${p_{\mathrm{T}}}>30$ GeV) for $|\eta |<2.5$ ($2.5 <|\eta |< 4.5$).

Jets containing *b*-hadrons (*b*-jets) are identified (*b*-tagged) using the MV1c algorithm, which is an improved version of the MV1 algorithm [23] with higher rejection of jets containing *c*-hadrons (*c*-jets). It combines in a neural network the information from various algorithms based on track impact-parameter significance or explicit reconstruction of secondary decay vertices. The operating point of this algorithm chosen for this analysis has an efficiency of about 70 % for *b*-jets in $t\bar{t}$ events and a *c*-jet (light-jet) mis-tag rate less than 20 % (1 %).

Lepton (electron or muon) candidates are identified in two categories: loose and tight, in order of increasing purity. Electron candidates are reconstructed from energy clusters in the electromagnetic calorimeter matched to reconstructed tracks in the inner tracking system. They are identified using likelihood-based methods [24, 25]. Loose electrons must satisfy “very loose likelihood” identification criteria and are required to have ${p_{\mathrm{T}}}>7$ GeV and $|\eta |<2.47$. Tight electrons are selected from the loose electrons and must also satisfy the “very tight likelihood” identification criteria. Muon candidates are reconstructed using information from the inner tracker and the muon spectrometer [26]. Loose muons are required to have ${p_{\mathrm{T}}}>7$ GeV and $|\eta |<2.7$. Tight muons are then selected from the loose muons, by requiring ${p_{\mathrm{T}}}>25$ GeV and $|\eta |<2.5$. They must be reconstructed in both the muon spectrometer and the inner tracker. For the loose leptons, the scalar sum of the transverse momenta of tracks within a cone of size $\Delta R = \sqrt{(\Delta \phi )^2+(\Delta \eta )^2} = 0.2$ around the lepton candidate, excluding its own track, is required to be less than 10 % of the transverse momentum of the lepton. For the tight leptons, there are more stringent isolation requirements: the sum of the calorimeter energy deposits in a cone of size $\Delta R = 0.3$ around the lepton candidate, excluding the energy associated with it, must be less than 4 % of the lepton candidate energy, and the track-based isolation requirement is tightened from 10 to 4 %.

The missing transverse momentum vector, ${{{\varvec{E}}_{\mathrm{T}}^{\mathrm{miss}}}}$, is computed using fully calibrated and reconstructed physics objects, as well as clusters of calorimeter-cell energy deposits that are not associated with any object [27]. Only calibrated jets with ${p_{\mathrm{T}}}$ greater than 20 GeV are used in the computation. The jet energy is also corrected for pile-up effects [28]. A track-based missing transverse momentum vector, ${{{\varvec{p}}_{\mathrm{T}}^{\mathrm{miss}}}}$, is calculated as the negative vector sum of transverse momenta of reconstructed tracks associated with the primary vertex and within $|\eta |<2.5$.

Monte Carlo (MC) simulated samples are produced for both the signal and background processes. Unless otherwise stated, the simulation [29] is performed using the ATLFAST-II package [30], which combines a parameterized simulation of the ATLAS calorimeter with the Geant4-based [31] full simulation for the rest of the subdetector systems.

Signal events from $q\bar{q}'\rightarrow VH$ with $H\rightarrow \text {inv.}$ are produced using the NLO Powheg method as implemented in the Herwig++ generator [32]. The $gg\rightarrow ZH$ production process contributes approximately 5 % to the total *ZH* cross section. Events from the $gg\rightarrow ZH$ production process are not simulated, but are taken into account by increasing the $q\bar{q}\rightarrow ZH$ cross section as a function of the Higgs boson ${p_{\mathrm{T}}}$ by the appropriate amount. The gluon-fusion signal events are produced using the Powheg generator interfaced to Pythia8 for parton showering and hadronization. The production of $q\bar{q}'\rightarrow VH$ followed by the SM $H\rightarrow b\bar{b}$ decay is considered as a background for the search. The Pythia8 generator is used to produce these events. The cross sections of all Higgs production processes are taken from Ref. [33].

A significant source of background is the production of $V+$jets and of $t\bar{t}$ events. A sample of $V+$jets events is generated using the Sherpa generator [34] with massive *b*- and *c*-quarks. Events from the $t\bar{t}$ process are generated using the Powheg generator interfaced with Pythia6 [35]. Other background contributions include diboson ($WW, WZ\ \mathrm{and}\ ZZ$) and single top-quark production. The Powheg generator interfaced to Pythia8 is used to produce diboson events. The diboson cross sections are calculated at NLO in QCD using the MCFM program [36] with the MSTW2008NLO parton distribution functions (PDFs) [37]. The *s*-channel and *Wt* single top-quark events are produced using the Powheg generator, as for $t\bar{t}$ production. The remaining *t*-channel process is simulated with the AcerMC generator [38] interfaced to Pythia6. Cross sections of the three single top-quark processes are taken from Refs. [39–41]. Table 1 summarizes the MC generators, PDFs and normalization cross sections used in this analysis.

**Table 1:** List of MC generators, parton distribution functions (PDFs) and cross sections used for the signal and background processes. The $H\rightarrow \text {inv.}$ signal cross sections are given for $m_H = 125$ GeV and assume SM production and BR($H\rightarrow \text {inv.}$) $=$ 100 %. Details are given in the text

Process	Generator	PDFs	Cross section (pb)
$t\bar{t}$	Powheg $+$ Pythia	CT10 [42]	Normalized to data
*V*+jets	Sherpa	CT10	Normalized to data
Single top			
*t*-channel	AcerMC	CTEQ6L1 [43]	88
*s*-channel	Powheg $+$ Pythia	CT10	5.6
*Wt*	Powheg $+$ Pythia	CT10	22
Diboson			
*WW*	Powheg $+$ Pythia	CT10	52
*WZ*	Powheg $+$ Pythia	CT10	9.2
*ZZ*	Powheg $+$ Pythia	CT10	3.3
SM VH			
$q\bar{q}'\rightarrow VH(\rightarrow b\bar{b})$	Pythia	CTEQ6L1	0.18
$gg\rightarrow ZH(\rightarrow b\bar{b})$	Powheg $+$ Pythia	CT10	0.0038
Signals			
$q\bar{q}\rightarrow Z(\rightarrow jj) H(\rightarrow \text {inv.})$	Herwig++	CT10	0.29
$q\bar{q}'\rightarrow W(\rightarrow jj) H(\rightarrow \text {inv.})$	Herwig++	CT10	0.48
$gg\rightarrow H(\rightarrow \text {inv.})$	Powheg $+$ Pythia	CT10	19

## Event selection

Events are required to pass an ${E_{\mathrm{T}}^{\mathrm{miss}}}$ trigger with a threshold of 80 GeV, which is a cut applied at the third level. The ${E_{\mathrm{T}}^{\mathrm{miss}}}$ trigger is fully efficient for ${E_{\mathrm{T}}^{\mathrm{miss}}}>160$ GeV and 97 % efficient for ${E_{\mathrm{T}}^{\mathrm{miss}}}=120$ GeV. An efficiency correction is derived from $W\rightarrow \mu \nu$+jets and $Z\rightarrow \mu ^{+}\mu ^{-}$+jets events. This correction is below 1 % for 120 GeV$<{E_{\mathrm{T}}^{\mathrm{miss}}}<160$ GeV. Events are also required to have ${E_{\mathrm{T}}^{\mathrm{miss}}}>120$ GeV, ${p_{\mathrm{T}}^{\mathrm{miss}}}>30$ GeV, no loose leptons and two or three “signal jets” (satisfying $|\eta |<2.5$, ${p_{\mathrm{T}}}>20$ GeV and leading jet ${p_{\mathrm{T}}}>45$ GeV). The inclusion of 3-jet events improves the signal efficiency. A requirement is made on ${{H}_{\mathrm{T}}}$, defined as the scalar sum of the ${p_{\mathrm{T}}}$ of all jets: ${{H}_{\mathrm{T}}}>120\ (150)$ GeV for events with two (three) jets. This cut is employed to avoid a trigger bias introduced by the dependence of the trigger efficiency on the jet activity, as also discussed in Ref. [44]. Events are discarded if they have additional jets with ${p_{\mathrm{T}}}>20~(30)$ GeV and $|\eta |<2.5$ ($2.5<|\eta |<4.5$) to reduce the contribution from the $t\bar{t}$ background process.

For *VH* signal events, ${E_{\mathrm{T}}^{\mathrm{miss}}}$ resulting from the $H\rightarrow \text {inv.}$ decay is expected to be strongly correlated with the transverse momentum of the vector boson *V* (${p_{\mathrm{T}}^V}$). Since the ${E_{\mathrm{T}}^{\mathrm{miss}}}$ distribution of the signal is harder than that of the background, additional sensitivity in the analysis is gained by optimizing the selection cuts separately for four ${E_{\mathrm{T}}^{\mathrm{miss}}}$ ranges. Here and in the following, the dijet refers to the two leading jets in events with three jets. The dijet invariant mass, $m_{jj}$, is required to be consistent with that of the *W* / *Z* boson. In addition a requirement on the radial separation between the two jets, $\Delta R_{jj}$, is made as the jets are expected to be close in for highly boosted *V*-bosons. Both the $m_{jj}$ and the $\Delta R_{jj}$ cuts reduce the *V*+jets and the $t\bar{t}$ backgrounds, and depend on ${E_{\mathrm{T}}^{\mathrm{miss}}}$. The cut values are given in Table 2.

Multijet events are copiously produced in hadron collisions. Fluctuations in jet energy measurements in the calorimeters can create ${E_{\mathrm{T}}^{\mathrm{miss}}}$ in these events and therefore mimic the signal. To suppress their contribution, additional selection criteria are applied to the azimuthal angles between ${{{\varvec{E}}_{\mathrm{T}}^{\mathrm{miss}}}}$, ${{{\varvec{p}}_{\mathrm{T}}^{\mathrm{miss}}}}$ and jets: $\Delta \phi ({{{\varvec{E}}_{\mathrm{T}}^{\mathrm{miss}}}},{{{\varvec{p}}_{\mathrm{T}}^{\mathrm{miss}}}})<\pi /2$, $\min [\Delta \phi ({{{\varvec{E}}_{\mathrm{T}}^{\mathrm{miss}}}},\mathrm{jet})]>1.5$ and $\Delta \phi ({{{\varvec{E}}_{\mathrm{T}}^{\mathrm{miss}}}},\mathrm{dijet})>2.8$. Here $\Delta \phi ({{{\varvec{E}}_{\mathrm{T}}^{\mathrm{miss}}}},{{{\varvec{p}}_{\mathrm{T}}^{\mathrm{miss}}}})$ is the azimuthal angle between ${{{\varvec{E}}_{\mathrm{T}}^{\mathrm{miss}}}}$ and ${{{\varvec{p}}_{\mathrm{T}}^{\mathrm{miss}}}}$, $\min [\Delta \phi ({{{\varvec{E}}_{\mathrm{T}}^{\mathrm{miss}}}},\mathrm{jet})]$ the angle between ${{{\varvec{E}}_{\mathrm{T}}^{\mathrm{miss}}}}$ and its nearest jet, and $\Delta \phi ({{{\varvec{E}}_{\mathrm{T}}^{\mathrm{miss}}}},\mathrm{dijet})$ is the angle between ${{{\varvec{E}}_{\mathrm{T}}^{\mathrm{miss}}}}$ and the momentum vector of the dijet system. These requirements are based on characteristics of events with mismeasured ${E_{\mathrm{T}}^{\mathrm{miss}}}$ in the multijet background, while taking advantage of the expected topologies of signal events.

Finally, the selected events are further categorized according to *b*-tag multiplicity (zero, one and two *b*-tagged jets) to improve the sensitivity. Combined with the two categories in jet multiplicity (two and three jets), there are in total six categories in the signal region.

**Table 2:** The ${E_{\mathrm{T}}^{\mathrm{miss}}}$-dependent event selections of the signal region for the four ${E_{\mathrm{T}}^{\mathrm{miss}}}$ ranges

${E_{\mathrm{T}}^{\mathrm{miss}}}$ range (GeV)	120–160	160–200	200–300	$>$300
Variable	Selection			
$\Delta R_{jj}$, 2- and 3-jet events	0.7–2.0	0.7–1.5	$<$1.0	$<$0.9
$m_{jj}$, 2-jet events (${\,\hbox {GeV}}$)	70–100	70–100	70–100	75–100
$m_{jj}$, 3-jet events (${\,\hbox {GeV}}$)	50–100	55–100	60–100	70–100

## Background estimation

In addition to the signal region, a number of control regions, designed to estimate various background contributions, are defined. They include the signal sideband (events not passing the $m_{jj}$ requirement), and the regions dominated by *V*+jets and $t\bar{t}$ events as discussed below. The multijet background is estimated from the data. The distributions of the *V*+jets and $t\bar{t}$ backgrounds are taken from MC simulation while their normalizations are estimated from the data. The remaining diboson, single-top and SM VH(bb) backgrounds are obtained from MC simulation.

The multijet background is estimated using four regions defined by requirements on $\Delta \phi ({{{\varvec{E}}_{\mathrm{T}}^{\mathrm{miss}}}},{{{\varvec{p}}_{\mathrm{T}}^{\mathrm{miss}}}})$ and $\min [\Delta \phi ({{{\varvec{E}}_{\mathrm{T}}^{\mathrm{miss}}}},\mathrm{jet})]$, as listed in Table 3. The shapes of the $m_{jj}$ and ${E_{\mathrm{T}}^{\mathrm{miss}}}$ distributions in the signal region A are taken from region C and the normalizations are determined by the ratio of the numbers of events in regions B and D.

**Table 3:** Definition of the signal region, A, and the three regions B, C and D used to estimate the multijet background in the signal region

Region	A	B	C	D
$\Delta \phi ({{{\varvec{E}}_{\mathrm{T}}^{\mathrm{miss}}}},{{{\varvec{p}}_{\mathrm{T}}^{\mathrm{miss}}}})$	$<\!\pi /2$	$<\!\pi /2$	$>\!\pi /2$	$>\!\pi /2$
$\min [\Delta \phi ({{{\varvec{E}}_{\mathrm{T}}^{\mathrm{miss}}}},\mathrm{jet})]$	$>$1.5	$<$0.4	$>$1.5	$<$0.4

The normalizations of the *V*+jets backgrounds are estimated using control regions enhanced in *W*+jets and *Z*+jets events. In all cases at least one lepton is required to have ${p_{\mathrm{T}}}>25$ GeV. The *W*+jets events are selected by requiring exactly one tight lepton, ${E_{\mathrm{T}}^{\mathrm{miss}}}>20$ GeV (${E_{\mathrm{T}}^{\mathrm{miss}}}>50$ GeV if ${p_{\mathrm{T}}^W}>200$ GeV), exactly two signal jets and ${m_{\mathrm{T}}^W}<120$ GeV.^3^ Moreover, ${p_{\mathrm{T}}^W}>100$ GeV is required in order to approximately match the phase space of the signal region. The *Z*+jets events are selected by requiring two loose leptons of the same flavour with opposite charges with invariant mass $83<m_{\ell \ell }<99$ GeV, at least two signal jets and a dilepton transverse momentum greater than 100 GeV. The kinematic distributions of the *V*+jets backgrounds are obtained from simulation that takes into account the different flavour composition of the jets. The simulated events are reweighted depending on the $\Delta \phi (\text {jet}_1,\text {jet}_2)$ and ${p_{\mathrm{T}}^V}$ to better match the data distributions [44]. The *Z*+jets control region has a small contribution from $t\bar{t}$ (1.3 %), which is estimated using a $t\bar{t}$ control region. This region is selected by requiring events to have two oppositely charged leptons of different flavour (one of which has ${p_{\mathrm{T}}}>25$ GeV) and passing the loose selection requirements, and at least two signal jets which are *b*-tagged. The signal sideband and the *V*+jets control regions are divided to match the categorization of the signal region while the $t\bar{t}$ control region remains as one category as described above. For the *V*+jets and $t\bar{t}$ control regions, the distributions of the multijet background are obtained from control regions defined by inverting the lepton isolation requirement and the normalizations are determined by template fits [44].

## Systematic uncertainties

The experimental systematic uncertainties considered include the trigger efficiency, object reconstruction and identification efficiency, and object energy and momentum scales as well as resolutions. Among these, the jet energy scale (JES) and resolution (JER) uncertainties have the largest impact on the result. The JES uncertainties are $\pm$3 and $\pm$1 % for central jets with a ${p_{\mathrm{T}}}$ of 20 GeV and 1 TeV, respectively. The JER uncertainty varies from between $\pm$10 and $\pm$20 %, depending on the pseudorapidities of the jets, for jets with ${p_{\mathrm{T}}}=20$ GeV to less than $\pm$5 % for jets with ${p_{\mathrm{T}}}>200$ GeV. The JER and JES uncertainties are also propagated to the ${E_{\mathrm{T}}^{\mathrm{miss}}}$ uncertainty. The *b*-tagging uncertainty depends on jet ${p_{\mathrm{T}}}$ and comes mainly from the uncertainty on the measurement of the efficiency in $t\bar{t}$ events [23]. The dominant contribution arises from jets matched to *b*-hadrons in the MC record of the particles’ true identities. Their efficiency uncertainties are at the level of $\pm$2–3 % over most of the jet ${p_{\mathrm{T}}}$ range, but reach $\pm$5 % for ${p_{\mathrm{T}}}=20$ GeV and $\pm$8 % above ${p_{\mathrm{T}}}=200$ GeV [45]. The uncertainty on the integrated luminosity is $\pm$2.8 %. It is derived following the same methodology as that detailed in Ref. [46].

For the backgrounds, a large number of modelling systematic uncertainties are considered, which account for possible differences between the data and the MC models. These uncertainties are estimated following the studies of Ref. [44] and are briefly summarized here. The uncertainties on the *V*+jets backgrounds come mainly from the knowledge of jet flavour composition and the ${p_{\mathrm{T}}^V}$, $\Delta \phi _{jj}$ and ${m_{jj}}$ distributions. For $t\bar{t}$ production, uncertainties on the top quark transverse momentum and the $m_{jj}$, ${E_{\mathrm{T}}^{\mathrm{miss}}}$ and ${p_{\mathrm{T}}^V}$ distributions are considered. The diboson background uncertainties are dominated by the theoretical uncertainties of the cross-section predictions, which include contributions from the renormalization and factorization scales and the choice of PDFs. The robustness of the multijet background estimation is assessed by varying the definition of the control regions B and D and an uncertainty of $\pm$100 % is assigned for this small background ($<$1 % in the signal regions).

The uncertainty on the signal acceptance is evaluated by changing the factorization and renormalization scale parameters, parton distribution function choices and the parton shower choices. For the *VH* signal, the dominant uncertainty is from parton shower modelling, which can be as large as $\pm$8 %. For the $gg\rightarrow H$ signal, the dominant uncertainty originates from the renormalization and factorization scales and can be as large as $\pm$15 % in the high ${E_{\mathrm{T}}^{\mathrm{miss}}}$ regions. Additional corrections to the Higgs boson ${p_{\mathrm{T}}}$ distribution of the $gg\rightarrow H$ signal are applied to match the distribution from a calculation at NNLO$+$NNLL provided by HRes2.1 [47, 48]. The detailed precedures are following the ones used in the $H\rightarrow \gamma \gamma$ and $H\rightarrow WW^{*}$ analyses as described in Ref. [49, 50]. The related uncertainties are also taken into account.

## Results

**Table 4:** Predicted and observed numbers of events for the six categories in the signal region. The yields and uncertainties of the backgrounds are shown after the profile likelihood fit to the data. In this fit all categories share the same signal-strength parameter. The quoted uncertainties combine the statistical and systematic contributions. These can be smaller for the total background than for individual components due to anti-correlations. The yields and uncertainties of the signals are shown as expected before the fit for $m_H=125$ GeV and BR$(H\rightarrow \text {inv.})=100\,\%$. Signal contributions from VBF and $t\bar{t}H$ production are estimated to be negligible

*b*-tag category	0-tag	1-tag	2-tag
Process	2-jet events		
Background			
*Z*+jets	24400 $\pm$ 1100	1960 $\pm$ 200	164 $\pm$ 13
*W*+jets	20900 $\pm$ 770	1160 $\pm$ 130	47 $\pm$ 7
$t\bar{t}$	403 $\pm$ 74	343 $\pm$ 65	57 $\pm$ 10
Single top	149 $\pm$ 16	107 $\pm$ 14	11 $\pm$ 2
Diboson	1670 $\pm$ 180	227 $\pm$ 25	64 $\pm$ 7
SM VH(*bb*)	1.5 $\pm$ 0.5	6 $\pm$ 2	3 $\pm$ 1
Multijet	26 $\pm$ 43	8 $\pm$ 7	0.7 $\pm$ 0.9
Total	47560 $\pm$ 490	3804 $\pm$ 64	347 $\pm$ 15
Signal			
$gg\rightarrow H$	403 $\pm$ 95	25 $\pm$ 6	2.1 $\pm$ 0.5
$W(\rightarrow jj) H$	425 $\pm$ 45	44 $\pm$ 6	0.6 $\pm$ 0.1
$Z(\rightarrow jj) H$	217 $\pm$ 19	42 $\pm$ 4	26 $\pm$ 2
Data	47404	3831	344

	3-jet events		
Background			
*Z*+jets	9610 $\pm$ 580	795 $\pm$ 93	53 $\pm$ 7
*W*+jets	7940 $\pm$ 510	479 $\pm$ 70	21 $\pm$ 4
$t\bar{t}$	443 $\pm$ 53	437 $\pm$ 53	63 $\pm$ 7
Single top	97 $\pm$ 14	66 $\pm$ 9	6.4 $\pm$ 0.9
Diboson	473 $\pm$ 54	55 $\pm$ 6	13 $\pm$ 2
SM VH(*bb*)	0.8 $\pm$ 0.3	2.6 $\pm$ 0.9	1.4 $\pm$ 0.5
Multijet	22 $\pm$ 29	4 $\pm$ 4	0.6 $\pm$ 0.6
Total	18580 $\pm$ 200	1840 $\pm$ 40	158 $\pm$ 7
Signal			
$gg\rightarrow H$	224 $\pm$ 55	15 $\pm$ 4	1.2 $\pm$ 0.5
$W(\rightarrow jj) H$	110 $\pm$ 16	11 $\pm$ 1	0.14 $\pm$ 0.03
$Z(\rightarrow jj) H$	65 $\pm$ 7	12 $\pm$ 1	6.1 $\pm$ 0.7
Data	18442	1842	159

The potential $H\rightarrow \text {inv.}$ signal is extracted through a combined likelihood fit to the observed ${E_{\mathrm{T}}^{\mathrm{miss}}}$ distributions of the signal region and its sideband and the ${p_{\mathrm{T}}^V}$ distributions of the control regions (${p_{\mathrm{T}}^V}$ is defined as ${p_{\mathrm{T}}^W}$, ${p_{\mathrm{T}}^Z}$ and ${p_{\mathrm{T}}^{e+\mu}}$ for the *W*+jets, *Z*+jets and $t\bar{t}$ control regions, respectively). The normalizations of the *V*+jets and $t\bar{t}$ backgrounds are free parameters in this fit. The ${E_{\mathrm{T}}^{\mathrm{miss}}}$ distributions are binned in such a way that each bin yields approximately the same amount of expected signal. The 2-jet categories of the signal region are split into ten bins, while fewer bins are used in the 3-jet categories and the sideband. Most *V*+jets control regions are split into five ${p^{V}_{\mathrm{T}}}$ bins, each yielding approximately the same amount of expected background. The 0-tag category of the *V*+jets control regions and the $t\bar{t}$ control region are used inclusively in the fit. The signal strength $\mu$, defined as the ratio of the signal yield ($\sigma _{VH} \times \text {BR}(H\rightarrow \text {inv.})$) relative to the SM production cross section and assuming BR$(H\rightarrow \text {inv.})=100\,\%$, is used to parameterize the signal in the data. A binned likelihood function is constructed as the product of Poisson probability terms comparing the numbers of events observed in the data to those expected from the assumed signals and estimated background contributions for all categories of the signal and control regions. The likelihood takes into account the background normalization and the systematic uncertainties. It is maximized to extract the most probable signal-strength value, $\hat{\mu }$.

Table 4 shows the numbers of observed events in the data compared to the numbers of estimated background events from the likelihood fit for each signal category. In all categories the data agrees with the background estimation. The backgrounds are dominated by *Z*+jets and *W*+jets events. Subleading backgrounds come from top and diboson production. The SM *VH* and multijet background contributions are very small with the final event selection.

**Fig. 1:**
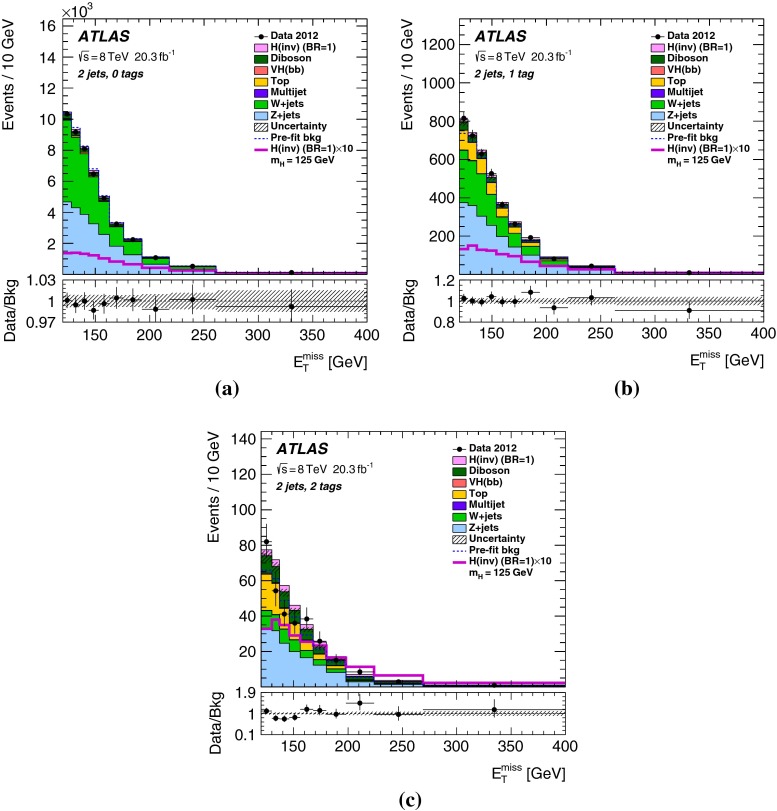
The missing transverse momentum (${E_{\mathrm{T}}^{\mathrm{miss}}}$) distributions of the 2-jet events in the signal region for the **a** 0-*b*-tag, **b** 1-*b*-tag and **c** 2-*b*-tag categories. The data are compared with the background model after the likelihood fit. The *bottom plots* show the ratio of the data to the total background. The signal expectation for $m_H = 125$ GeV and BR$(H\rightarrow \text {inv.})=100\,\%$ is shown on *top of the background* and additionally as an *overlay line*, scaled by the factor indicated in the *legend*. The total background before the fit is shown as a *dashed line*. The *hatched bands* represent the total uncertainty on the background

**Fig. 2:**
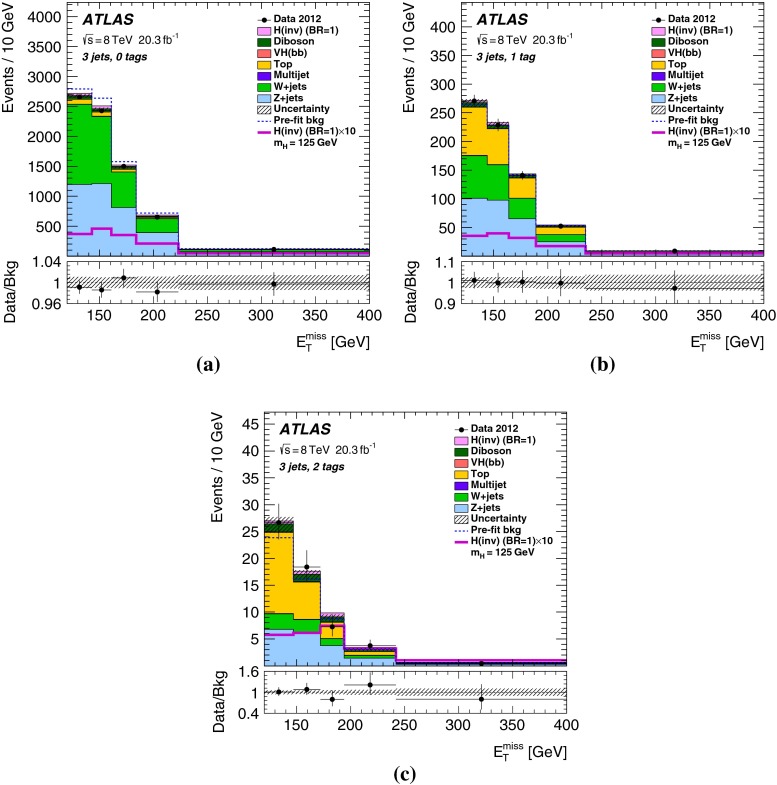
The missing transverse momentum (${E_{\mathrm{T}}^{\mathrm{miss}}}$) distributions of the 3-jet events in the signal region for the **a** 0-*b*-tag, **b** 1-*b*-tag and **c** 2-*b*-tag categories. The data are compared with the background model after the likelihood fit. The *bottom plots* show the ratio of the data to the total background. The signal expectation for $m_H = 125$ GeV is shown on *top of the background* and additionally as an *overlay line*, scaled by the factor indicated in the *legend*. The total background before the fit is shown as a *dashed line*. The *hatched bands* represent the total uncertainty on the background

**Fig. 3:**
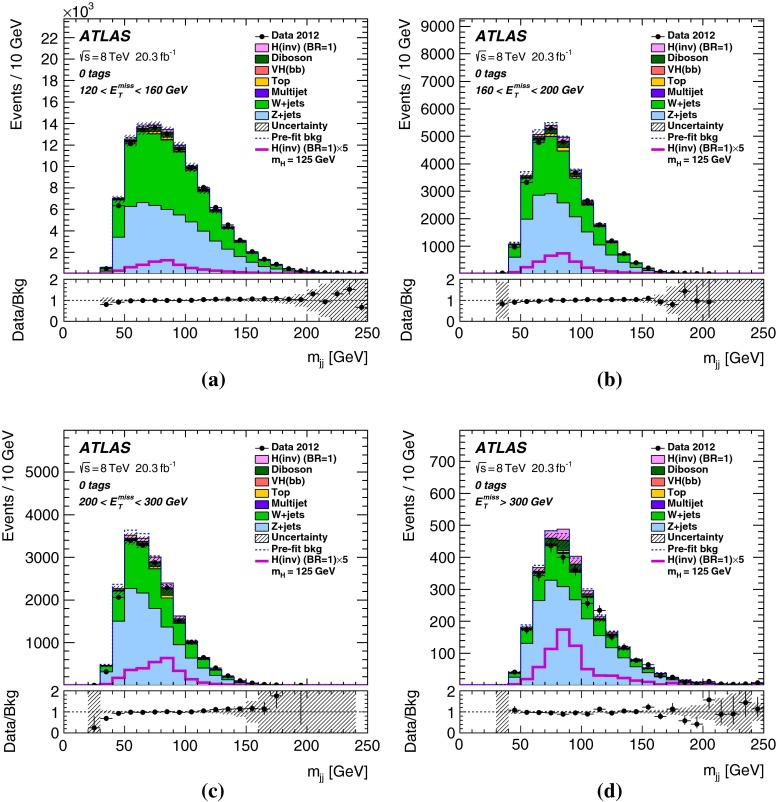
The dijet invariant mass ($m_{jj}$) distributions in the signal region for the 0-*b*-tag category, for events with ${E_{\mathrm{T}}^{\mathrm{miss}}}$ in the range **a** (120–160 GeV), **b** (160–200 GeV), **c** (200–300 GeV) and **d** ($>$300 GeV). The data are compared with the background model after the likelihood fit. The *bottom plots* show the ratio of the data to the total background. The signal expectation for $m_H = 125$ GeV is shown on *top of the background* and additionally as an *overlay line*, scaled by the factor indicated in the *legend*. The total background before the fit is shown as a *dashed line*. The *hatched bands* represent the total uncertainty on the background

**Fig. 4:**
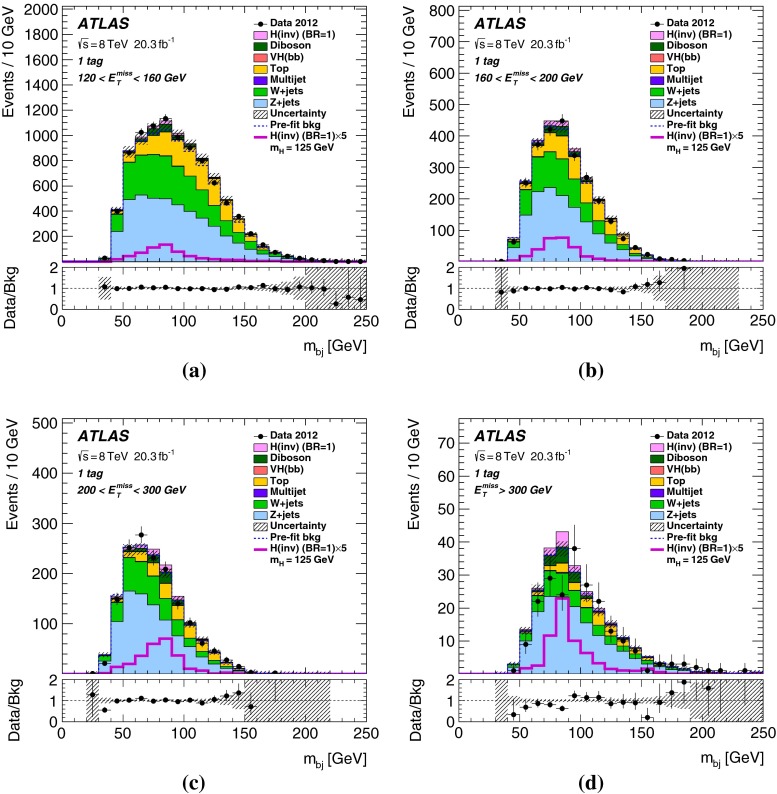
The dijet invariant mass ($m_{bj}$) distributions in the signal region for the 1-*b*-tag category, for events with ${E_{\mathrm{T}}^{\mathrm{miss}}}$ in the range **a** (120–160 GeV), **b** (160–200 GeV), **c** (200–300 GeV) and **d** ($>$300 GeV). The data are compared with the background model after the likelihood fit. The *bottom plots* show the ratio of the data to the total background. The signal expectation for $m_H = 125$ GeV is shown on *top of the background* and additionally as an *overlay line*, scaled by the factor indicated in the *legend*. The total background before the fit is shown as a *dashed line*. The *hatched bands* represent the total uncertainty on the background

**Fig. 5:**
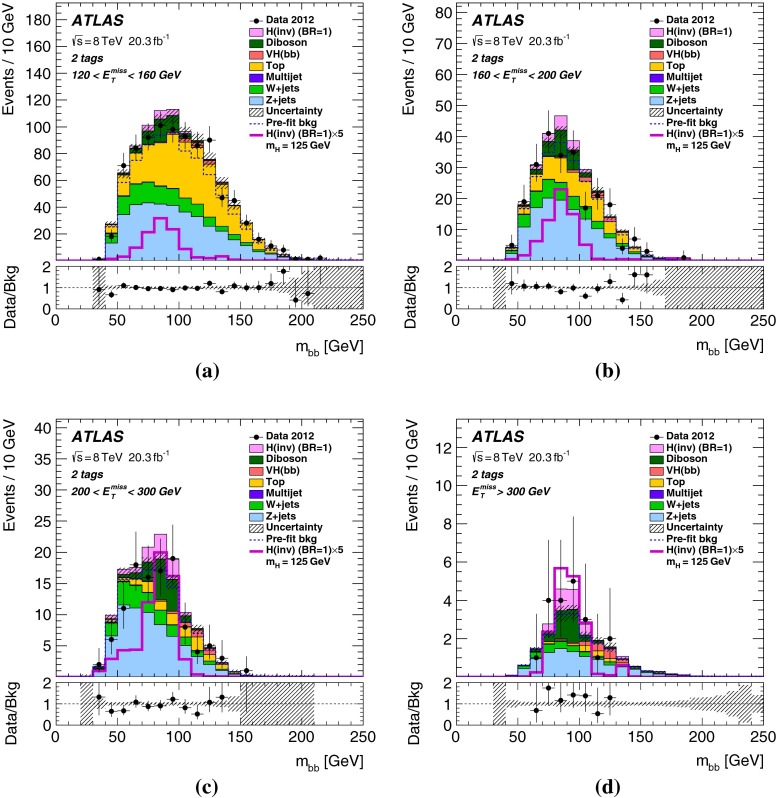
The dijet invariant mass ($m_{bb}$) distributions in the signal region for the 2-*b*-tag category, for events with ${E_{\mathrm{T}}^{\mathrm{miss}}}$ in the range **a** (120–160 GeV), **b** (160–200 GeV), **c** (200–300 GeV) and **d** ($>$300 GeV). The data are compared with the background model after the likelihood fit. The *bottom plots* show the ratio of the data to the total background. The signal expectation for $m_H = 125$ GeV is shown on *top of the background* and additionally as an *overlay line*, scaled by the factor indicated in the *legend*. The total background before the fit is shown as a *dashed line*. The *hatched bands* represent the total uncertainty on the background

The fit reveals no significant excess of events over the background expectations and yields a best-fit signal-strength value of $\hat{\mu } = -0.13^{+0.43}_{-0.44}$, which is consistent with zero. The contributions from the individual systematic uncertainties are summarized in Table 5. The systematic uncertainty sources which have the largest impacts are the energy scale of the jets and of ${E_{\mathrm{T}}^{\mathrm{miss}}}$ along with the modelling (shape and normalization) of the diboson and *V*+jets backgrounds. The ${E_{\mathrm{T}}^{\mathrm{miss}}}$ distributions of the events passing the signal region selection are shown in Figs. 1 and 2 after the profile likelihood fit to the data. The fit results are also propagated to the $m_{jj}$ distributions of the events passing the signal region selection (without the $m_{jj}$-window cuts). The corresponding plots are shown in Figs. 3, 4 and 5 for the three *b*-tag categories separately.

**Table 5:** Impacts of sources of systematic uncertainty on the uncertainty of the fitted signal strength, $\Delta \mu$, in the data. Only sources with contributions larger than $\pm$0.03 are listed

Source	Impact on $\Delta \mu$	
Object systematic uncertainties		
Jets & ${E_{\mathrm{T}}^{\mathrm{miss}}}$	$+$0.22	$-$0.22
Luminosity	$+$0.04	$-$0.03
*b*-tagging	$+$0.05	$-$0.04
Background systematic uncertainties		
Diboson	$+$0.26	$-$0.29
*Z*+jets	$+$0.21	$-$0.22
*W*+jets	$+$0.15	$-$0.16
$t\bar{t}$	$+$0.06	$-$0.05
Multijet	$+$0.07	$-$0.07
Total		
Total systematic uncertainty	$+$0.41	$-$0.43
Data statistical uncertainty	$+$0.12	$-$0.12
Total uncertainty	$+$0.43	$-$0.44

**Fig. 6:**
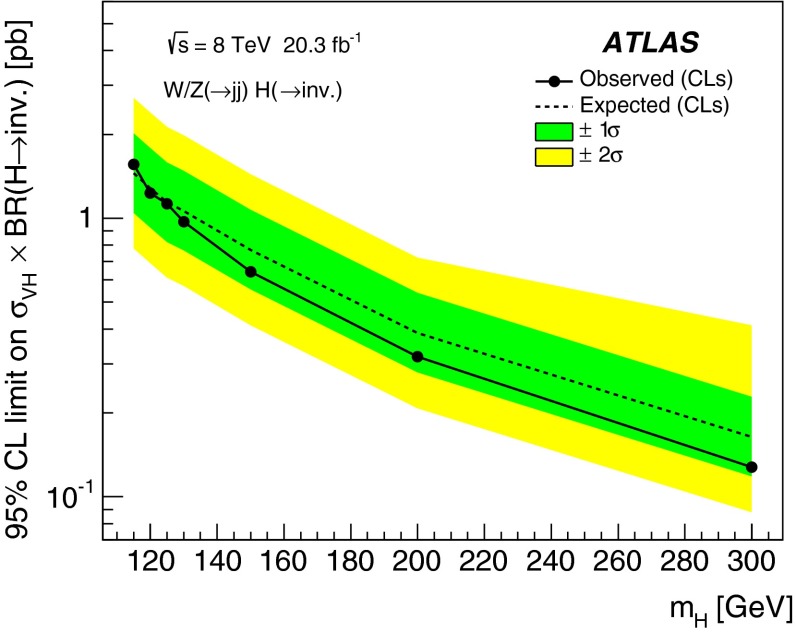
Upper limits on $\sigma _{VH} \times \text {BR}(H\rightarrow \text {inv.})$ at 95 % CL for a Higgs boson with $115 <m_H< 300$ GeV. The full and *dashed lines* show the observed and expected limits, respectively

The null results are used to set 95 % confidence level (CL) upper limits on the product of the *VH* cross sections and the $V \rightarrow jj$ and $H\rightarrow \text {inv.}$ decay branching ratio, $\sigma _{VH} \times \text {BR}(H\rightarrow \text {inv.})$, as a function of the Higgs boson mass in the range $115<m_H<300$ GeV as shown in Fig. 6. The limits are computed with a modified frequentist method, also known as CL$_s$ [51], and a profile-likelihood-based test statistic [52]. At $m_H=125$ GeV, for *VH* production, a limit of 1.1 pb is observed compared with 1.1 pb expected. These combined results for *VH* production assume the SM proportions of the *WH* and *ZH* contributions. Observed (expected) limits are also derived for the two contributions separately, 1.2 (1.3) pb for *WH* and 0.72 (0.59) pb for *ZH*. As shown in Table 4, the 2-tag categories are almost only sensitive to *ZH*, the 1-tag categories are equally sensitive to *WH* and *ZH*, and the 0-tag categories are more sensitive to *WH* production. The two processes contribute approximately equally to the sensitivity.

For the discovered Higgs boson at $m_H=125$ GeV, an observed (expected) upper limit of 78 % (86 %) at 95 % CL on the branching ratio of the Higgs boson to invisible particles is set. These limits are derived assuming SM production and combining contributions from *VH* and gluon-fusion processes. The gluon-fusion production process contributes about 39 % (29 %) to the observed (expected) combined sensitivity.

## Summary

In summary, Higgs boson decays to particles that are invisible to the ATLAS detector are searched for in the final states of two or three jets and large missing transverse momentum in a *pp* collision dataset corresponding to an integrated luminosity of 20.3 ${{\mathrm {fb}}^{-1}}$ at a centre-of-mass energy of 8 TeV. No excess of events over the expected backgrounds is observed. The results are used to constrain the cross section for *VH* production followed by the decay $H\rightarrow \text {inv.}$ for $115<m_H<300$ GeV. The observed 95 % CL upper limit on $\sigma _{VH} \times \text {BR}(H\rightarrow \text {inv.})$ varies from 1.6 pb at 115 GeV to 0.13 pb at 300 GeV. Assuming SM production and including the $gg\rightarrow H$ contribution, an observed (expected) upper limit of 78 % (86 %) on BR$(H\rightarrow \text {inv.})$ is derived for the discovered Higgs boson with $m_H=125$ GeV. This independent result is comparable to that of the ATLAS *ZH* search with $Z\rightarrow \ell \ell$ and $H\rightarrow \text {inv.}$ [19].

## Footnotes

1 The ATLAS experiment uses a right-handed coordinate system with its origin at the nominal interaction point (IP) in the centre of the detector and the *z*-axis along the beam pipe. The *x*-axis points from the IP to the centre of the LHC ring, and the *y*-axis points upward. Cylindrical coordinates $(r,\phi )$ are used in the transverse plane, $\phi$ being the azimuthal angle around the *z*-axis. The pseudorapidity is defined in terms of the polar angle $\theta$ as $\eta =-\ln [\tan (\theta /2)]$. Transverse momenta are computed from the three-momenta, $\varvec{p}$, as ${p_{\mathrm{T}}}= |\varvec{p}| \sin \theta$.

2 The primary vertex is taken to be the reconstructed vertex with the highest $\Sigma {p_{\mathrm{T}}}^{2}$ of the associated tracks.

3 The transverse mass, ${m_{\mathrm{T}}^W}$, is calculated from the transverse momentum and the azimuthal angle of the charged lepton, ${p_{\mathrm{T}}^{\ell }}$ and $\phi ^{\ell }$, and from the missing transverse momentum’s magnitude, ${E_{\mathrm{T}}^{\mathrm{miss}}}$, and azimuthal angle, $\phi ^{\mathrm{miss}}$: ${m_{\mathrm{T}}^W}= \sqrt{2{p_{\mathrm{T}}^{\ell}}{E_{\mathrm{T}}^{\mathrm{miss}}}(1-\cos (\phi ^{\ell }-\phi ^{\mathrm{miss}}))}$. The transverse momentum of the *W* boson, ${p_{\mathrm{T}}^W}$, is reconstructed as the magnitude of the vector sum of the lepton transverse momentum and the ${{{\varvec{E}}_{\mathrm{T}}^{\mathrm{miss}}}}$.
